# TLR9 signalling activation via direct ligation and its functional consequences in CD4 + T cells

**DOI:** 10.1111/sji.13214

**Published:** 2022-09-11

**Authors:** Ravi Kumar Sharma, Jyoti Sharma, Rajendra Kumar, Darshan Badal, Ajinkya Pattekar, Shobha Sehgal, Amod Gupta, Pooja Jain, Naresh Sachdeva

**Affiliations:** ^1^ Advanced Eye Centre Post Graduate Institute of Medical Education and Research (PGIMER) Chandigarh India; ^2^ Department of Microbiology and Immunology and the Institute for Molecular Medicine and Infectious Disease Drexel University College of Medicine Philadelphia Pennsylvania USA; ^3^ Division of Biological Sciences Indian Institute of Science Education and Research Mohali Punjab India; ^4^ Department of Endocrinology Post Graduate Institute of Medical Education and Research (PGIMER) Chandigarh India; ^5^ Department of Immunopathology Post Graduate Institute of Medical Education and Research (PGIMER) Chandigarh India; ^6^ Division of Rheumatology, Department of Medicine Karolinska Institutet Solna Sweden; ^7^ Department of Oncology Johns Hopkins University School of Medicine Baltimore Maryland USA

**Keywords:** CD4+ T cells, effector T cells, immune suppression, oligodeoxynucleotides, T cell proliferation, TLR9

## Abstract

CpG Oligodeoxynucleotides (ODNs) are established TLR9 ligands; however, their functional responses in CD4+ T cells are believed to be independent of TLR9 and MyD88. We studied ligand‐receptor interactions of ODN 2216 and TLR9 in human CD4+ T cells and assessed their consequences in terms of TLR9 signalling and cell phenotype. We demonstrated that the uptake of ODN 2216, a synthetic TLR9 agonist, is controlled by TLR9 signalling molecules and results in an increase in the expression of TLR9 signalling molecules, regulated via a feedback mechanism. Next, the uptake of ODN 2216 resulted in TLR9 signalling dependent but MyD88 independent increase in expression of TGF‐β. Finally, ODN 2216 treated CD4+ T cells showed an anti‐inflammatory phenotype that was similar to Th3 type of regulatory T cells. These Th3‐like cells were able to suppress the proliferation of untreated CD4+ T cells. Collectively, our results demonstrate a direct and interdependent relationship between ODN 2216 uptake and TLR9 signalling in CD4+ T cells. Our findings thus pave the way for future research to explore direct modulation of adaptive immune cells, using innate immune ligands, to subvert exaggerated inflammatory responses.

## 1 INTRODUCTION

Toll like receptor 9 (TLR9) is an innate immune sensing receptor that recognizes cytosine‐phosphate‐guanosine (CpG) oligodeoxynucleotides (ODNs) and responds by secretion of proinflammatory cytokines.^1^, ^2^, ^3^ Innate immune cells like dendritic cells and macrophages recruit myeloid differentiation primary response gene 88 (MyD88) and co‐localize with CpG ODNs in an endocytosis‐dependent fashion.^2^, ^4^ Uptake of ODNs leads to signalling via recruitment of molecules like interleukin 1 receptor associated kinase 1 (IRAK1), IRAK4, TNF receptor associated factor 3 (TRAF3), TRAF6 and interferon regulatory factor 7 (IRF7), culminating in IFN‐α production.^5^ TLR9‐mediated stimulation of B cells and plasmacytoid dendritic cells (pDCs) has been shown to induce Th1 immune responses and antitumor activity in animal models and patients.^6^ ODNs have also been tried in experimental models as immunosuppressive agents to treat allergy,^7^ inflammation^8^ and autoimmunity.^9^, ^10^ Further, ODNs may confer opposing effects on pDCs, resulting in the dominance of IL‐23 and TGF‐β in low and high doses respectively.^11^ Reports have shown that post alum adsorbed CpG ODN challenge in an ovalbumin model of allergic lung inflammation resulted in MyD88 and IL‐10 dependent inhibition of Th2 response without polarization towards Th1 response.^12^ ODN 2216 stimulation has been shown to increase IL‐10 secretion^13^ and decrease IL‐6 expression in B cells.^14^ Also, the ligation of ODN 2216 in pDCs has been demonstrated to result in the induction of CD4 + CD25+ regulatory T cells (Tregs).^15^ In addition, stimulation of human pDCs with TLR7 and TLR9 ligands has been shown to induce CD8+ LAG‐3 + FoxP3 + CTLA4+ Tregs, which have indoleamine‐pyrrole‐2,3‐dioxygenase 1 (IDO1) dependent immunosuppressive activity.^11^, ^16^

While the expression and function of TLR9 and ligand‐receptor relationship for ODN and TLR9 in innate immune cells are evident, there are scattered reports on the expression of TLR9 in cells of the adaptive immune system, especially CD4+ T cells.^17^, ^18^, ^19^, ^20^, ^21^ ODNs are known to increase IL‐2 secretion and proliferation of CD4+ T cells upon concomitant T cell receptor (TCR) ligation, but the involvement of TLR9 signalling in the process is debated.^22^, ^23^, ^24^ One study has shown this secretion to be dependent on MyD88, protein kinase B (Akt) and phosphoinositide 3‐kinase (PI‐3 K) molecules,^23^ whereas another has reported the increase in proliferation to be independent of TLR9 and MyD88.^24^ A study on Helios expressing CD4+ Tregs demonstrates that TLR9 engagement on these cells results in their enhanced stabilization during in vitro expansion.^25^ These studies suggest that ODNs can have a direct effect on CD4+ T cell proliferation, independent of antigen presenting cells (APCs). However, an active role for TLR9 signalling is still unclear in CD4 + T cells. We have shown that the TLR9 ligand ODN 2216, gets internalized by endocytosis and interacts with lysosomal associated membrane protein‐1 (LAMP‐1) in CD4+ T cells^20^ and its uptake in CD4+ T cells activates TLR9 signalling. However, to the best of our knowledge, no study has looked at the ligand‐receptor relationship between TLR9 and ODNs and the role of TLR9 signalling in the downstream effects of ODN uptake in CD4+ T cells, particularly in CD4+ T cells in the absence of APCs. This is particularly important in wake of those inflammatory diseases, where TLR ligands can directly influence the ensuing adaptive immune responses as observed by us and other groups.^21^, ^26^, ^27^

In the present study, we demonstrated that the process of ODN 2216 uptake in CD4+ T cells and activation of the TLR9 signalling pathway are interdependent events regulated via a feedback control mechanism. Activation of TLR9 signalling also exerts an impact on functional characteristics of CD4+ T cells including proliferation and cytokine response. While TLR9 signalling increases the proliferative response of engaged CD4 + T cells, such cells can control the proliferation of unexposed CD4 + T cells and act like Th3 type of Tregs under in‐vitro conditions. Our study collectively provides a detailed analysis of direct ODN 2216 uptake induced functional changes and TLR9 signalling pathway in CD4+ T cells. This can have far reaching implications in various inflammatory diseases where CD4 + T cells are directly exposed to an inflammatory milieu rich in TLR ligands.

## 2 MATERIALS AND METHODS

### 2.1 Subject recruitment and sample collection

Twelve healthy laboratory control subjects (aged 25‐35 years) were recruited from the Department of Ophthalmology, Post Graduate Institute of Medical Education and Research (PGIMER) Chandigarh, after prior informed consent. The study was duly approved by PGIMER Chandigarh institute ethics committee (No's PGI/IEC/2011/532‐533/24/11/11 and NK/1385/PhD/2484/07/09/17). This study was executed in accordance with the recommendations of the Institutional ethics committee of PGIMER. All subjects gave written informed consent in accordance with the Declaration of Helsinki. Fifteen millilitres of peripheral blood was withdrawn from all subjects participating in the study and used for isolation of peripheral blood mononuclear cells (PBMCs) using lymphoprep™ density gradient (Stem cell technologies, Vancouver, Canada).

### 2.2 Magnetic cell isolation

CD4 + CD25‐T (mentioned as CD4+ T cells in the manuscript for convenience unless otherwise specified) cells were purified from the PBMCs using a CD4 + CD25+ T cell isolation kit based on a two‐step negative isolation protocol, as per the manufacturer's prescription (Stem cell technologies). The kit separates CD4 + CD25+ and CD4 + CD25‐ T cells using positive and negative selection respectively. We collected negatively selected cell population and assessed cellular purity by immunophenotyping using anti‐human‐CD3 Alexa Fluor 700 (Clone OKT3), CD4 APC‐Cy7 (clone RPA‐T4), CD25 PE‐Cy7 (clone BC96) (Biolegend Inc., San Diego, CA, USA), and FoxP3‐BD Horizon V450 (Clone 236A/E7) (BD) antibodies on a flow cytometer, (LSR™ Fortessa, BD Biosciences, San Jose, CA, USA). Purity of isolated cells (CD3 + CD4 + CD25‐FoxP3‐) ranged between 90‐98% (Figure S1A‐C). All data were acquired on the same flow cytometer using FACS Diva™ software version 7.0 (BD Biosciences) and analysis was done by FACS Diva™ or Flowjo™ version 10.5.3 9.0 (Flowjo LLC, Ashland, OR, USA).

### 2.3 TLR9 agonist uptake assay

Uptake of the TLR9 agonist, ODN 2216 FITC (Invivogen, San Diego, CA, USA) was assessed in magnetically sorted CD4+ T cells using flow cytometry. Briefly, 10^5^ cells were exposed to 500 ng/mL of the ligand overnight (14 hours) and the uptake of ODN 2216 was evaluated on the basis of a shift in fluorescence per cell as median fluorescence intensity (MFI) (Figure S1D). GpC ODN 2243 was used as a control to assess whether the effects are specific to CpG ODN 2216. For experiments involving assessment of uptake, ODN 2243 was labelled using Ulysis™ Alexa Fluor 488 nucleic acid labelling kit as per manufacturer instructions (Thermofisher Scientific Inc., Waltham, MA). Cells without labelled ODN were used as negative control (NC) to see baseline fluorescence in the FITC channel in each setup. Dead cells can non‐specifically bind to ODNs including ODN 2216 (Figure S1E). In view of this, all further analysis was performed on live cells by tight gating on scatter gate, followed by gating of 7‐AAD dye (e‐bioscience) negative events, for all experiments which did not require permeabilization (Figure S1). On an average, 98‐99% of cells after forward and side scatter tight gating were live cells. Further analysis was done on live cells only.

### 2.4 Gene expression studies

Impact of ODN 2216 ligation on activation of the TLR9 signalling pathway was assessed by studying mRNA expression of major TLR9 signalling pathway genes including, TLR9, MyD88, IRAK1, IRAK4, TRAF3, TRAF6, IRF7, IRF3, TGF‐β activated kinase 1 (TAB1), Akt, inhibitor of nuclear factor kappa‐B kinase subunit alpha (IKK‐α or CHUK), inhibitor of nuclear factor kappa b kinase subunit beta (IKBKB), nuclear factor kappa‐light‐chain‐enhancer of activated B cells (NF‐kB) and other candidates (Table S1) using a customized mRNA expression PCR array (Qiagen, Hilden, Germany). GAPDH and beta actin were used as housekeeping controls (Table S1). Fold change values were calculated using ΔΔ Ct method and compared to the unstimulated controls. The gene expression of these molecules was studied before and after the stimulation of CD4+ T cells with ODN 2216. The expression of genes upstream of IRF7 was further confirmed in 6‐12 controls by quantitative real‐time RT‐PCR using custom‐designed primers (Table S2). GAPDH was used as the endogenous housekeeping control gene for confirmation experiments. Briefly, total RNA was isolated from CD4+ T cells exposed to ODN 2216 using a TRI reagent™ solution (Ambion, Life Technologies, Waltham, MA, USA) and RNA was quantitated using a multimode reader (Biotek Inc, Winooski, VT, USA). One microgram of RNA was used for the synthesis of cDNA, at a final concentration of 10 ng/well. Briefly, the PCR conditions were; denaturation at 95 °C for 30 seconds, annealing at 60 °C for 1 minute, followed by extension at 72 °C for 30 seconds. All real‐time PCR samples were loaded in triplicates, and the mean Ct value was used for the calculation of fold change using ΔΔ C_t_ method. Sorted CD4+ T cells without ODN 2216 treatment were used as controls in all experiments. TLR9 antagonist, ODN TTAGGG treated cells were used as additional controls in these experiments.

### 2.5 siRNA transfection

To study the role of TLR9, TRAF3 and IRF7 in ODN 2216 uptake and subsequent events in CD4+ T cells, magnetically sorted CD4+ T cells were transfected separately with siRNA oligos directed against TLR9, TRAF3 and IRF7 (Eurogentec S.A, Seraing, Belgium). The optimum dosage of each siRNA molecule was standardized by assessing the viability of transfected cells using ***7***‐aminoactinomycin D (7‐AAD) (Biolegend). Briefly, 3.0 pg of oligo siRNA was mixed with 2 μl of mission**®** siRNA transfection reagent (Sigma), in 100 μl of serum free media (SFM) and incubated for 15 minutes at room temperature. This mix was then poured into a total of 10^5^ CD4+ T cells in fetal bovine serum (Thermofisher Scientific Inc., Waltham, MA, USA) containing media RPMI 1640 (Himedia Laboratories, Mumbai, India) in a 24‐well tissue culture plate. The cells were incubated at 37°C and 5% CO_2_ for 6 hours. The cells were washed with media and resuspended in 500 μl of T cell optimizer expansion SFM (Thermofisher Scientific Inc.) containing 150 IU/mL recombinant human IL‐2 (Peprotech, Rocky Hill, NJ) for another 18 hours. Cells transfected using a universal negative control siRNA (Sigma Aldrich) were used as a control in all siRNA‐mediated inhibition experiments, while those treated with siRNA transfection reagent alone were used as carrier controls. The efficiency of transfection was assessed by uptake of control siRNA in CD4+ T cells (Figure S1H). The silencing of TLR9 by siRNA was calculated by dividing MFIs for TLR9 after treatment with particular siRNA with an average of MFIs observed after control siRNA treatment and were compared between TLR9 siRNA silenced and control siRNA treated cells (Figure S1I). The silencing by TRAF3 and IRF7 siRNA was confirmed by real‐time RT‐PCR and there was a more than two‐fold decrease in mRNA expression of both molecules, as compared to control siRNA transfected cells (Figure S1). The same siRNA transfection protocol was followed for all experiments and siRNA‐treated cells will mean cells that underwent siRNA transfection using this protocol in the whole manuscript.

### 2.6 Inhibition of MyD88 and IRAK1

To assess the role of MyD88 and IRAK1 on ODN 2216 uptake and subsequent events in CD4+ T cells, sorted cells were treated with inhibitors of these molecules in separate sets of experiments. MyD88 inhibition was performed by treating 10^5^ CD4+ T cells with MyD88 inhibitory peptide (100 μM) (Novus Biologicals, Littleton, CO) for 24 hours.^28^ Similarly, IRAK1 (IRAK1/4 inhibitor I, 300 nM) (Sigma)^29^ was inhibited by treating CD4+ T cells with indicated concentrations for 24 hours. The same inhibition protocol was followed in all experiments and inhibitor‐treated cells in the manuscript refer to cells exposed to the aforementioned treatment prior to use.

### 2.7 Intracellular TLR9 protein expression

The effect of ODN 2216 FITC uptake and inhibition of TLR9 signalling on the expression of TLR9 was also assessed by flow cytometry. Briefly, ODN 2216 treated CD4+ T cells were stained with pre‐titrated volumes of anti‐human CD3 Alexa Fluor (3 μl/test) and anti‐human CD4 allophycocyanin‐Cyanine7 (APC‐Cy7) (3 μl/test) in 100 μl PBS, followed by incubation for 20 minutes at room temperature in 5mlflow tubes. Tubes were washed with PBS and treated with Cytoperm Cytofix as per the manufacturer's recommendations (BD). Cells were then stained with intracellular anti‐human TLR9 APC (clone eB72‐1665) (ebiosciences, San Diego, CA, USA) and incubated for 30 minutes at room temperature. The cells were finally washed twice with PBS before acquisition on the LSR™ Fortessa flow cytometer (BD). Anti‐rat IgG2a kappa APC antibody was used as the isotype control, while fluorescence minus one was used as a control for gating positive cells. As cellular controls, TLR9 expression was also confirmed in monocytes and B cells (positive controls) and Raji cell line (B‐cell line), using anti‐human TLR9 APC antibody.

### 2.8 CD4+ T cell proliferation assay

Carboxyfluorescein succinimidyl ester (CFSE) (Sigma) or eFluor 670 (Thermofisher Scientific) labelled sorted cells were cultured separately in presence of ODN 2216. TLR9 signalling candidates were inhibited with siRNA or inhibitors as mentioned previously, prior to the addition of ODN 2216 (Invivogen) in 48‐well culture plates in T cell optimizer expansion SFM (Thermofisher Scientific Inc.) containing recombinant human IL‐2 and Dynabeads™ human T cell activator cocktail (Thermofisher Scientific Inc.) for 3 days at 37°C, 5% CO_2_. Cells stimulated using Dynabeads™ alone were used as a control of cell proliferation, whereas cells stimulated using Dynabeads™ and ODN TTAGGG or ODN 2243 were used as control to validate the findings observed with ODN 2216. Proliferation index (PI) was calculated using the following formula, which has been followed previously as well.^21^

$$\text{Proliferation index}=\frac{\text{number of cells in}\;\mathrm{all}\;\text{generations}}{\text{number of original parent cells}}$$

where, the number of original parent cells $=\frac{p1}{1}+\frac{p2}{2}+\frac{p3}{4}+\frac{p4}{8}\ldots ..$

P1 = undivided cells, p2 = cells divided once, P3 = cells divided twice and so on.

### 2.9 Treg induction assay

To assess the impact of TLR9 ligation on induction of Tregs and expression of cytokines, 10^6^ sorted CD4+ T cells were cultured in presence of ODN 2216 FITC for 3 days using the reagents mentioned in T cell proliferation assay. TLR9 antagonist, ODN TTAGGG treated cells were used as an additional control in these experiments. Six hours before the termination of cultures, monensin (Sigma Aldrich) was added into all wells to block protein secretion. Induction of Tregs was evaluated on the basis of an increase in FoxP3 expression. Briefly, the exposed cells were stained using anti‐human CD3 AlexaFluor700, CD4 APC‐Cy7 and CD25 Phycoerythrin‐Cyanine7 (PE‐Cy7) antibodies followed by incubation at room temperature for 30 minutes. The cells were washed and permeabilized with FoxP3 staining buffer (Biolegend) as per the manufacturer's instructions. The permeabilized cells were stained intracellularly with anti‐human FoxP3‐BD Horizon V450 (BD), TGF‐β1 PE (clone TB7‐16B4) (e‐Bioscience), IL‐10 PE‐CF594 (clone JES3‐19F1) (BD), IFN‐γ brilliant violet 510 (BV510) (clone 4S.B3) (Biolegend), IL‐17A Peridinin chlorophyll protein (PerCP) (clone BL168) (Biolegend) and CTLA4 APC (clone L3D10). The effect of ODN 2216 stimulation on various parameters was evaluated and compared between stimulated and unstimulated cells. The frequency of Tregs was reported as CD4 + CD25 + FoxP3+ events as percent of CD4 + CD25+ T cells.

### 2.10 ELISA

The levels of secreted TGF‐β in CD4+ T cells stimulated with ODN 2216 were measured using a human TGF‐β ELISA kit as per manufacturer recommendations (e Bioscience).

### 2.11 T cell suppression assay

To assess the effect of ODN 2216 stimulated cells on proliferation of ODN 2216 untreated cells, T cell suppression assay was employed. CD4+ T cells were cultured in the presence ODN 2216 (500 ng/mL) for 2 days in CTS™ T cell optimizer expansion serum‐free medium containing 50 IU/mL of recombinant human IL‐2 (henceforth called suppressor cells). Cells were then washed twice with PBS pH 7.4 and cultured with eFluor 647‐labelled fresh CD4+ T cells (henceforth called responder cells) in varying ratios (4:1, 2:1, 1:2, 1:4). The cultures were finally terminated after 3 days. The proliferation index was calculated as mentioned previously.^21^ Percent suppression was then calculated at all ratios, by considering CD4+ T cells stimulated using a human T cell activator cocktail and IL‐2 alone as control of suppression. The formula for calculation of percentage suppression was described previously^30^ and followed by us earlier as well.^31^ Briefly,

$$\text{Percentage suppression}=\frac{\left(\mathrm{PI}\;\text{cells alone}-\mathrm{PI}\;\text{coculture}\right)}{\mathrm{PI}\;\text{cells alone}}x100$$

### 2.12 Statistical analysis

Data were presented as mean ± SD. Data were checked for normality using D'Agostino and Pearson omnibus normality test or KS normality test depending on sample size. The difference between various treatments was compared by repeated measures one‐way ANOVA or Friedman test, depending on the data distribution and multiple comparisons were tested using Dunn's multiple comparisons test, Dunnett's multiple comparison test or Sidak's multiple comparisons test. For difference with respect to control, normally distributed data were compared for using *t*‐test while data deviating from normality was compared using the Mann‐Whitney U test. All statistical analysis was performed using Graph Pad Prism™ 8.0 software and *P* values <0.05 were considered significant.

## 3 RESULTS

### 3.1 CD4 + T cells express TLR9 at a steady state

There have been an increasing number of reports on TLR9 expression and TLR responses in T cells under different conditions. To look for comparative expression of TLR9 in various immune cells, we performed intracellular TLR9 staining in representative sorted CD4 + T cells, THP‐1 cell line and buffy coat samples. Sorted CD4+ T cells also showed a clear shift in fluorescence (Figure S2A). THP‐1 cells also showed positive TLR9 staining (Figure S2B). The levels seemed to be similar between B cells (Figure S2C) and CD4 + T cells (Figure S2D). However, there seemed to be higher shifts in fluorescence for TLR9 fluorochrome for monocytes (Figure S2E,F) which was expected considering the lineage of monocytes as innate immune cells.

### 3.2 ODN 2216 stimulation activates TLR9 signalling in CD4+ T cells

**FIGURE 1:**
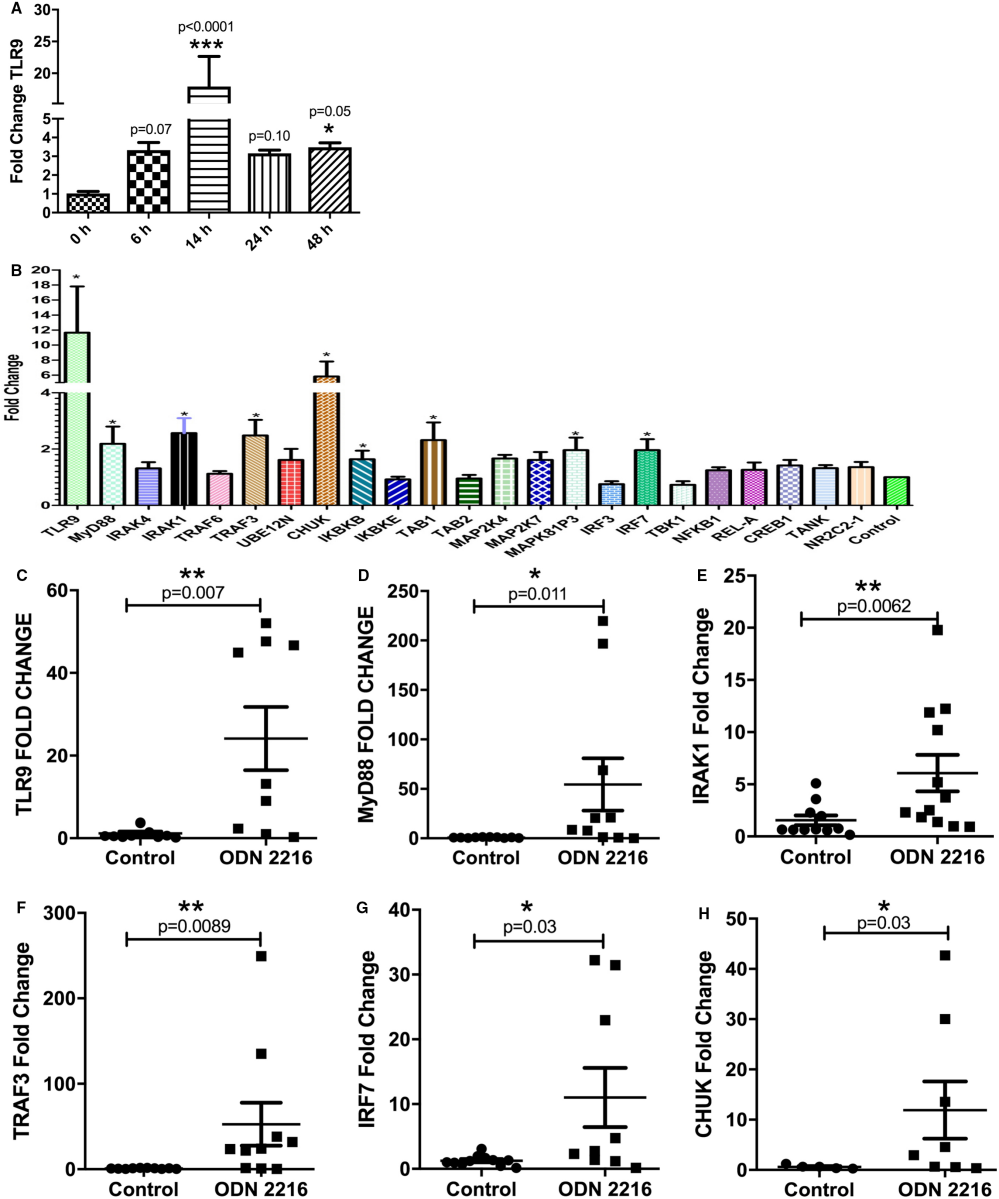
Effect of ODN 2216 uptake on activation of TLR9 signalling in CD4+ T cells. Isolated CD4+ T cells from six donors were cultured in presence of ODN 2216 (500 ng/mL) for varying time periods and mRNA expression of TLR9 was evaluated using real‐time RT‐PCR. (A) TLR9 expression kinetics was tested for the statistical difference using the Friedman test (*P* = .0007, *n* = 6), while multiple comparisons were made using Dunn's multiple comparisons test (*P* values shown in the figure). (B) Customized gene expression array was used for the assessment of activation of TLR9 signalling after ODN 2216 uptake, using beta actin and GAPDH as endogenous controls (Table S1). The results of PCR array were compared using one‐way ANOVA (*P* = .0001, *n* = 6). * represents genes showing significantly upregulated genes in TLR9 signalling pathway as assessed using Dunn's multiple comparisons test (*P* < .05) ** means *P* < .01 and *** means *P* < .001. (C‐K) The results of the array were confirmed using real‐time RT‐PCR for major genes of TLR9 signalling pathway. Each dot represents one donor in the individual graphs. Fold change values in figures C‐H were assessed for statistical difference using either t‐test {TLR9 (*P* = .007, *n* = 9), MyD88 (*P* = .011, *n* = 10)} or Mann‐Whitney test {IRAK1 (*P* = .006, *n* = 12), TRAF3 (*P* = .009, *n* = 10), IRF7 (*P* = .03, *n* = 9) and CHUK(*P* = .03, *n* = 8)}. Fold changes were calculated from the mean Ct values of 3 experimental replicates in each sample

We have recently shown that ODN 2216 is taken up intracellularly by CD4 + T cells via active endocytosis, which is inhibited by promethazine. To assess whether TLR9 ligand can activate TLR9 signalling, we evaluated TLR9 expression in ODN 2216 treated CD4+ T cells. A previous study had shown delayed TLR9 expression in B cells post ODN uptake.^32^ In view of this, we assessed the mRNA expression of TLR9 at various time points (6, 14 and 48 hr) after ODN 2216 treatment and observed increase in expression of TLR9, 6 hours (*P* = .07, *n* = 6) post uptake which sustained until 48 hours (*P* = .05, *n* = 6) (Figure 1A). Maximum levels of TLR9 mRNA were observed at 14 hours (*P* < .0001, *n* = 6) post addition of ODN 2216, and we chose this time point in all experiments. Next, we measured the expression of key molecules involved in TLR9 signalling in sorted CD4+ T cells, 14 hours post ODN 2216 uptake using a customized gene‐expression array. The expression of TLR9, MyD88, IRAK1, TRAF3, CHUK, IkBkB and IRF7 was significantly higher in ODN 2216 stimulated CD4+ T cells (*P* = .03, *n* = 6) (Figure 1B). The confirmatory qRT‐PCR in additional number of subjects also showed a significant increase in expression of TLR9 (*P* = .007, *n* = 9) (Figure 1C), MyD88 (*P* = .011, *n* = 10) (Figure 1D), IRAK1 (*P* = .006, *n* = 12) (Figure 1E), TRAF3 (*P* = .009, *n* = 10) (Figure 1F), IRF7 (*P* = .03, *n* = 9) (Figure 1G) and CHUK (*P* = .03, *n* = 8) (Figure 1H). To rule out non‐specific changes, we also assessed the expression of genes of TLR9 signalling after treatment of CD4+ T cells with TLR9 antagonist ODN TTAGGG. The expression of all genes was similar in antagonist‐treated and untreated cells (Figure S3A‐G). We also assessed the effect of ODN 2216 and control ODN 2243 on TLR9 protein expression by using polyclonal TLR9 antibodies. As expected, the uptake of ODN 2216 (*P* < .0001) (Figure S3H,I) but not ODN 2243 (*P* < .0001) (TLR7 agonist) (Figure S3J,K) resulted in increased TLR9 protein expression in CD4+ T cells. The results suggested that engagement of ODN 2216 in CD4+ T cells leads to activation of a TLR9 signalling pathway in CD4 + T cells.

### 3.3 TLR9 signalling molecules regulate the uptake of ODN 2216 in CD4+ T cells

**FIGURE 2:**
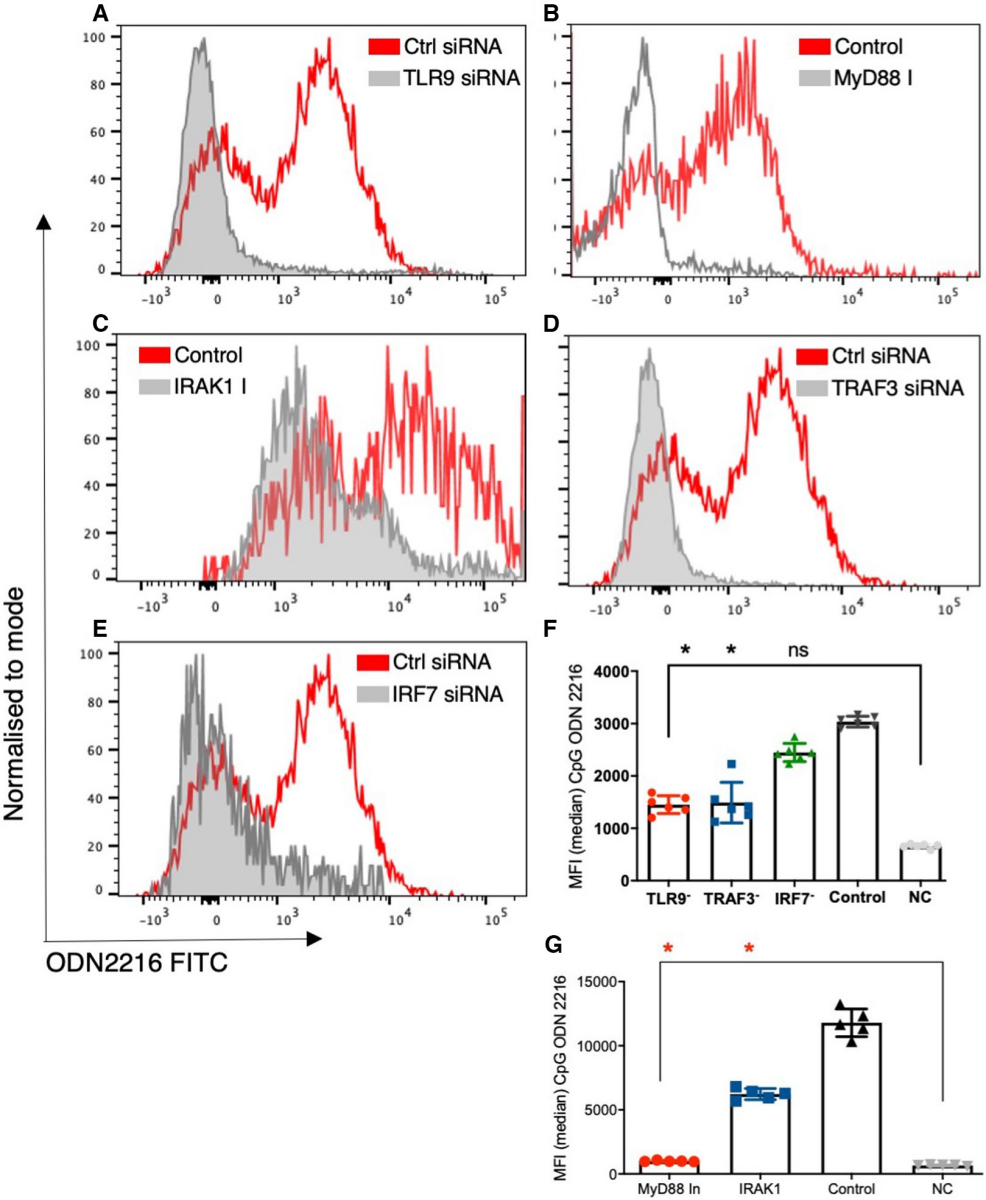
Effect of downstream signalling molecules on ODN 2216 uptake in CD4+ T cells. The uptake of ODN 2216 FITC was assessed in CD4+ T cells after inhibition of TLR9 signalling molecules. siRNA transfection was performed in CD4 + T cells from 6 individuals while MyD88 and IRAK inhibitors were tested in 5 individuals. (A‐E) Representative flow cytograms showing the extent of uptake of ODN 2216 in CD4 + T cells under different conditions (treatment and control). Control cells were cells cultured with ODN 2216 without any inhibitory treatment, while NC stands for negative control where no ODN 2216 was added. (F) Transfection using TLR9 (*P* = .03, *n* = 6), TRAF3 (*P* = .02, *n* = 6) but not IRF7 (*P* = .99, *n* = 6) siRNA showed significantly lower uptake of ODN 2216 as compared to control siRNA transfected cells from same individuals (*n* = 6, *P* < .0001). (G) MyD88 (*P* = .02, *n* = 5) and IRAK1 (*P* = .049, *n* = 5) inhibition resulted in significantly lower uptake of ODN 2216 (*P* < .0001, *n* = 5) Differences between groups were tested using Friedman test, while multiple comparisons were made Dunn's multiple comparisons test. Experiments with siRNA were performed in 6 different individuals while those with MyD88 and IRAK1 inhibitors were performed in 5 individuals. Each dot represents one individual

Next, we assessed the impact of TLR9 signalling on the uptake of the ligand in CD4+ T cells. We treated sorted CD4+ T cells with TLR9 siRNA, MyD88 inhibitor, IRAK1 inhibitor, TRAF3 siRNA, and IRF7 siRNA and studied the uptake of ODN 2216. There was a significant difference in uptake of ODN 2216 among different groups, upon inhibition of TLR9 (*P* = .0001, *n* = 5‐7) using siRNA transfection. We observed significant differences in uptake of ODN after inhibiting TLR9 signalling (*P* < .0001, *n* = 6) (Figure 2A‐F). We observed significantly lower uptake of ODN 2216 in CD4+ T cells treated with TLR9 siRNA as compared to those treated with control siRNA (*P* = .03, *n* = 6) (Figure 2A,F). Inhibition of MyD88 (*P* = .02, *n* = 5) (Figure 2B,G) and IRAK1 (*P* = .049, *n* = 5) (Figure 2C,G) using their respective inhibitors resulted in significantly lower uptake of ODN 2216 in CD4+ T cells as compared to control cells. Silencing of TRAF3 (*P* = .026, *n* = 6) (Figure 2D,F) but not IRF7 (*P* = .99, *n* = 6) (Figure 2E,F) resulted in lower uptake of ODN 2216 as compared to control siRNA treated cells. Importantly, the uptake was not completely abolished after inhibition as all inhibitor treated/transfected conditions showed higher MFI than the negative control. On the contrary, molecules involved in TLR9 signalling did not affect the uptake of control ODN i.e. ODN 2243 (Figure S4). These observations highlighted the role of TLR9 and MyD88 as adaptors for ODN 2216 in CD4+ T cells, already known for APCs.^33^, ^34^, ^35^

### 3.4 ODN 2216 activated TLR9 signalling pathway demonstrates a feedback control mechanism in CD4+ T cells

**FIGURE 3:**
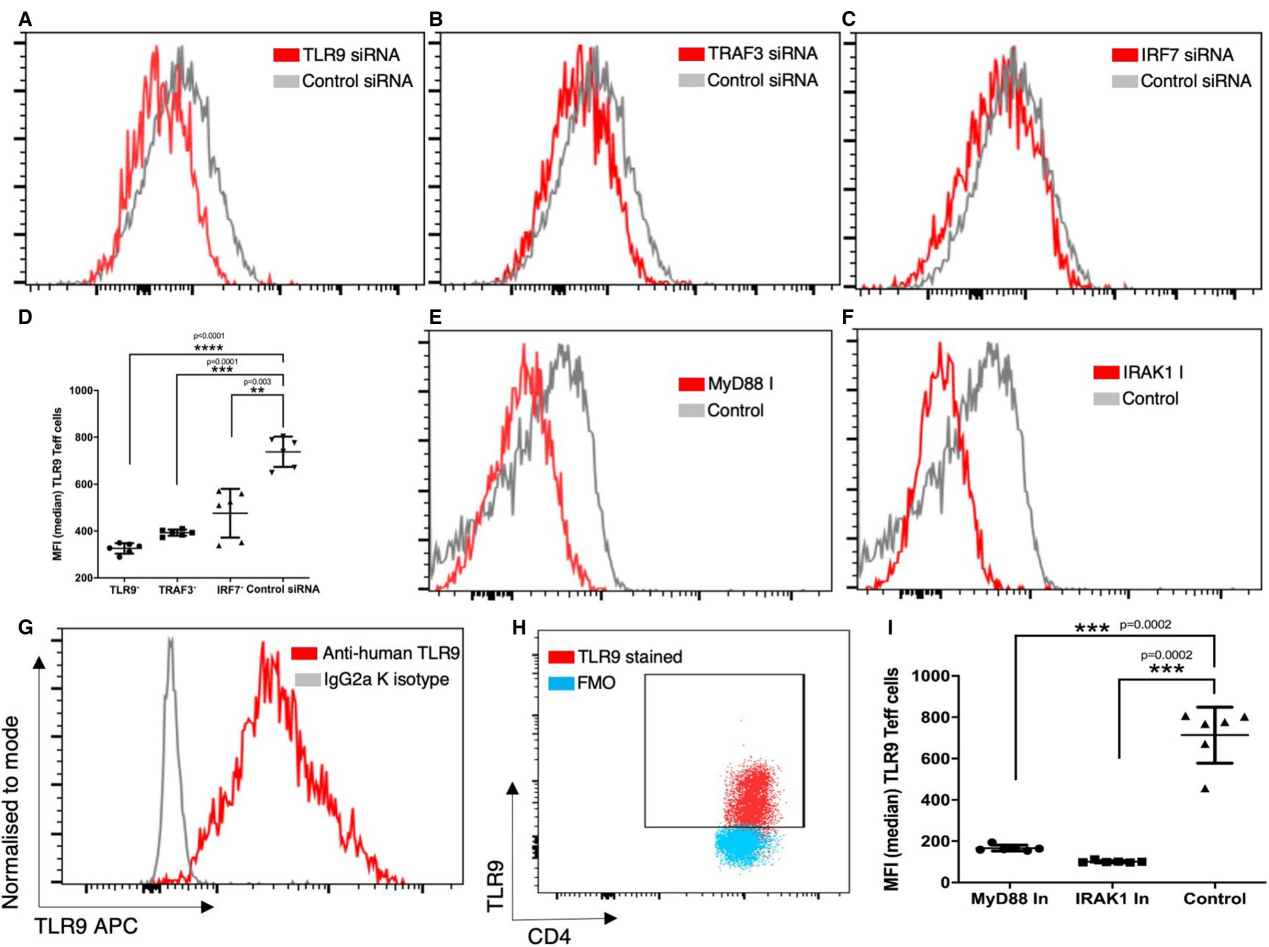
Effect of TLR9 signalling molecules on the expression of TLR9 in CD4+ T cells. The differences in expression of TLR9 upon inhibition of different signalling molecules were evaluated by flow cytometry using overlay graphs. (A‐C) Shows overlay flow cytograms containing a comparison of TLR9 expression upon treatment with inhibitory siRNA and control conditions. (D) TLR9 (*P* < .0001, *n* = 6), TRAF3 (*P* = .0001, *n* = 6) and IRF7 (*P* = .003, *n* = 6) siRNA transfection showed significantly lower TLR9 expression as compared to control siRNA transfected cells. The difference between groups was tested using repeated measures one‐way ANOVA, while Dunnett's multiple comparison test was used for post hoc tests. Cells were analysed for expression of TLR9 in cells treated with inhibitors of (E) MyD88 and (F) IRAK1 and compared with control cells. TLR9 staining was compared in reference to (G) isotype control antibody and (H) fluorescence minus one control tube. (I) Inhibition of MyD88 (*P* = .0002, *n* = 6) and IRAK1 (*P* = .0002, *n* = 6) resulted in significantly lower expression of TLR9 than control cells. The difference was tested using repeated measures one‐way ANOVA, while Dunnett's multiple comparisons test was used for post hoc tests. The *P* values depict approximated *P* values from multiple comparisons tests. Each dot represents data from one donor in the statistical graphs

Next, we inhibited different molecules of the TLR9 signalling pathway and assessed the expression of TLR9, post ODN 2216 stimulation in CD4+ T cells. There was a significant difference in TLR9 expression among different groups after inhibition of molecules (*P* < .0001, *n* = 6). We found a significant decrease in per cell expression of TLR9 after treatment with TLR9 siRNA (*P* < .0001, *n* = 6) (Figure 3A,D), TRAF3 siRNA (*P* = .0001, *n* = 6) (Figure 3B,D) and IRF7 siRNA (*P* = .003, *n* = 6) (Figure 3C,D) as compared to cells treated with control siRNA. Next, we observed a significant decrease in expression of TLR9 in CD4+ T cells upon inhibition of MyD88 (*P* = .0002, *n* = 6) (Figure 3E,I) and IRAK1 (*P* = .0002, *n* = 6) (Figure 3F,I) as compared to control cells (Figure 3G,H). TLR9 staining was confirmed in comparison to isotype control (Figure 3G) and fluorescence minus one control (Figure 3H) tubes. Collectively, our observations showed that downstream signalling molecules affect the expression of TLR9 after ODN 2216 uptake, highlighting the feedback‐dependent nature of the TLR9 signalling pathway in CD4+ T cells.

### 3.5 ODN 2216 ligation modulates the phenotype of CD4 + T cells:

**FIGURE 4:**
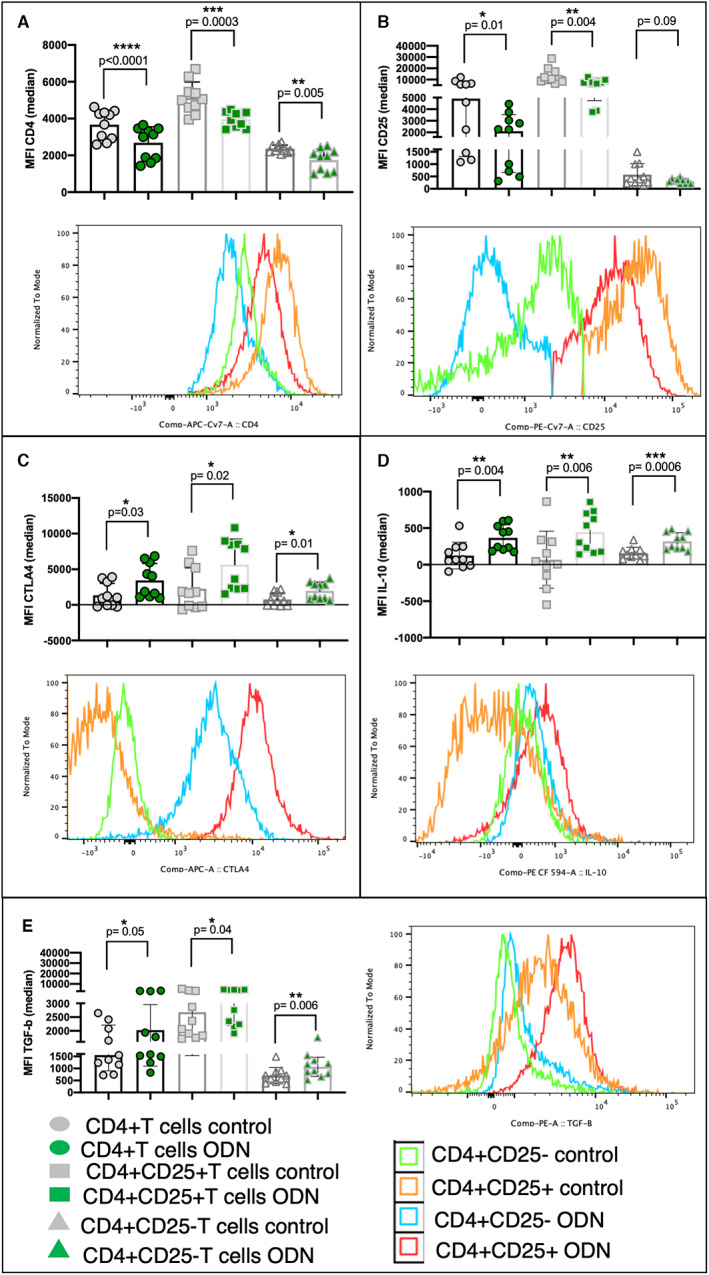
Effect of ODN 2216 ligation on modulation of CD4+ T cells. CD4+ T cells from ten healthy donors were cultured in presence of ODN 2216 for 3 days and the expression of various markers was assessed using cells treated with a human T cell activator cocktail as control. Overlay graphs were used to depict differences in the expression of various markers. (A‐E) Figures show the comparison of median fluorescence intensity in CD4 + T cells, CD4 + CD25 + T cells and CD4 + CD25‐T cells for different analytes including (A) CD4, (B) CD25, (C) CTLA4, (D) IL‐10 and (E) TGF‐β. (A) CD4 MFI in total CD4 + T cell population (*P* < .0001, *n* = 10), as well as CD4 + CD25+ (*P* = .0003, *n* = 10) and CD4 + CD25‐ T cells (*P* = .005, *n* = 10) was lower after ODN 2216 stimulation as compared to control. (B) A downregulation in CD25 expression was also observed in total CD4 + T cell population (*P* = .01, *n* = 10) and CD4 + CD25+ T cells (*P* = .004, *n* = 10). (C) ODN 2216 stimulation also resulted in an increase in expression of CTLA4 {in total CD4 + T cell population (*P* = .03, *n* = 10), CD4 + CD25 + T cells (*P* = .02, *n* = 10) and CD4 + CD25‐ T cells (*P* = .01, *n* = 10)}, (D) IL‐10 {(in total CD4 + T cell population (*P* = .004, *n* = 10), CD4 + CD25 + T cells (*P* = .006, *n* = 10) and CD4 + CD25‐ T cells (*P* = .0006, *n* = 10))} and (E) TGF‐β {(in total CD4 + T cell population (*P* = .05, *n* = 10), CD4 + CD25 + T cells (*P* = .04, *n* = 10) and CD4 + CD25‐ T cells (*P* = .006, *n* = 10))}. Each dot represents data from one donor in the individual graphs. Median fluorescence intensities of CD4, CD25, TGF‐β and IL‐10 were compared using paired *t* test, while that of CTLA4 was compared using the Wilcoxon matched‐pairs signed rank test

To assess the outcome of direct TLR9 ligation in CD4+ T cells, we evaluated the role of ODN 2216 ligation on the expression of various activation markers and cytokines. Isolated CD4 + T cells were analysed for expression of CD4, CD25, CTLA4, FoxP3, IL‐10, TGF‐β, IL‐17A, IFN‐γ. ODN 2216 stimulation resulted in a significant increase in the uptake of ODN 2216 uptake in the CD4 + T cell population, most of which was contributed by CD4 + CD25 + T cells (Figure S5A). The stimulation also resulted in a significant increase in proportion of CD4 + CD25+ T cells (*P* = .0035, *n* = 10), indicating activation of CD4 + T cells (Figure S5B). It was accompanied by a decrease in CD4 MFI in total CD4 + T cell population (*P* < .0001, *n* = 10), as well as CD4 + CD25+ (*P* = .0003, *n* = 10) and CD4 + CD25‐ T cells (*P* = .005, *n* = 10) (Figure 4A). Downregulation was less pronounced in CD4 + CD25‐T cells, consistent with previous observation on an increase in proportion of CD4 + CD25 + T cells. A downregulation in CD25 expression was also observed in total CD4 + T cell population (*P* = .01, *n* = 10) and CD4 + CD25+ T cells (*P* = .004, *n* = 10) (Figure 4B). However, we did not observe changes in expression of CD3 upon stimulation, suggestive of TCR independent mechanism of action (Figure S5C) and the same for effect on the expression of IFN‐γ (Figure S5D) or IL‐17A (Figure S5E) post‐ligation. ODN 2216 stimulation also resulted in an increase in the expression of CTLA4 (Figure 4C), IL‐10 (Figure 4D) and TGF‐β (Figure 4E) in all cell subsets. Notably, we did not observe any change in the expression of FoxP3 post‐ligation (Figure S5F). The cytokine expression pattern was specific to ODN 2216, as ODN TTAGGG treated cells had similar expression of TGF‐β (*P* = .25) (Figure S5G), CTLA4 (*P* = .31) (Figure S5H) and IL‐10 (*P* = .18) (Figure S5I) to that of untreated CD4+ T cells. Collectively, these results indicated a FoxP3‐independent induction of regulatory‐like phenotype in CD4 + T cells post ODN 2216 ligation.

### 3.6 ODN 2216 uptake‐induced TGF‐β expression in CD4+ T cells is dependent on TLR9 signalling

**FIGURE 5:**
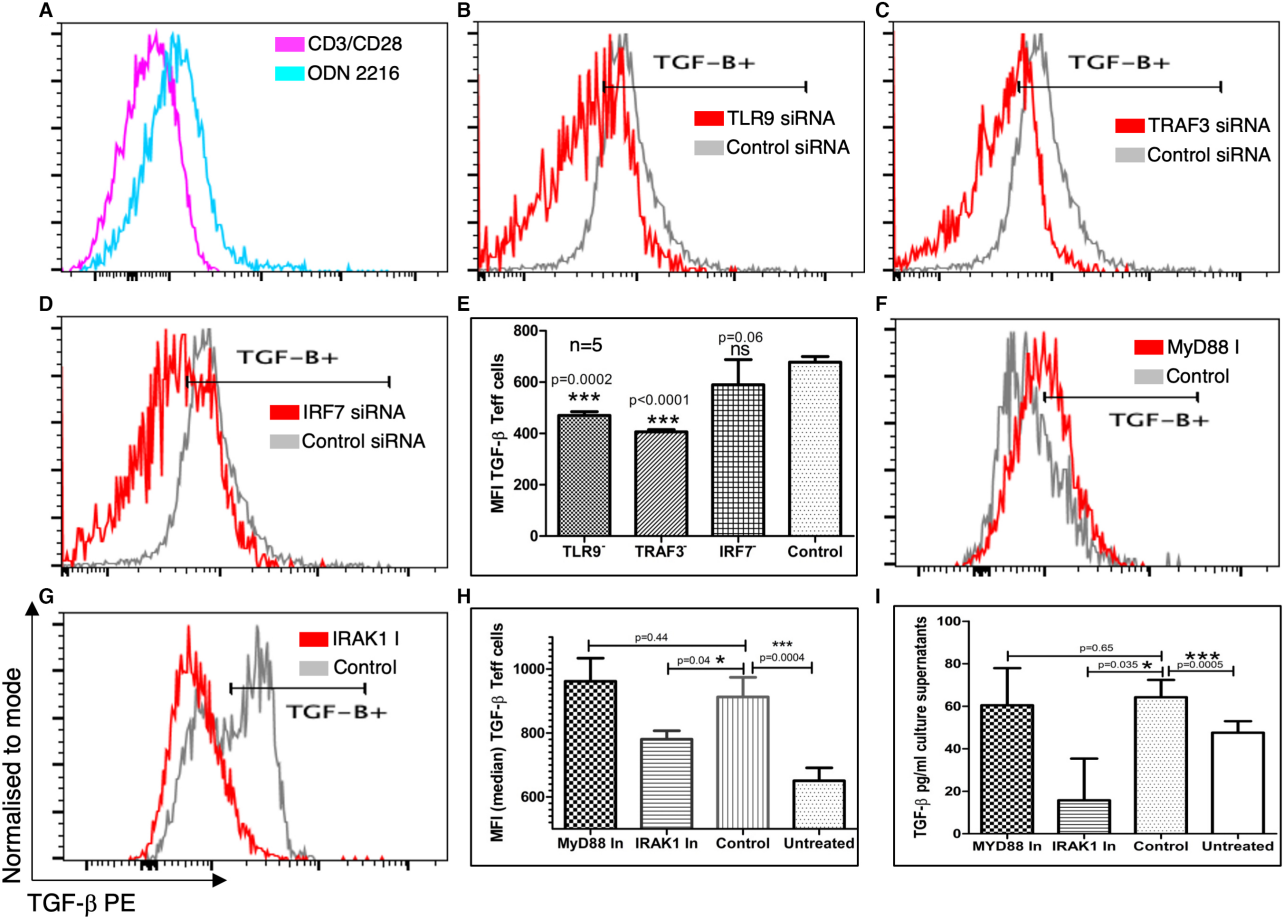
Effect of TLR9 signalling inhibition on TGF‐β expression in CD4+ T cells. CD4+ T cells were assessed for expression of intracellular TGF‐β1 using flow cytometry after treatment with inhibitors of the TLR9 signalling pathway or control conditions. (A) The intracellular levels of TGF‐β were measured by flow cytometry and compared to controls using overlay graphs. There was a marked decrease in TGF‐β expression after silencing of (B‐D) Overlay graphs showing expression of TGF‐β1 after silencing of TLR9, TRAF3 and IRF7 using siRNAs for respective molecules. (E) Statistical comparison of the effect of siRNA showed a significant decrease in intracellular expression of TGF‐β in CD4+ T cells, after treatment with TLR9 siRNA (*P* = .0004, *n* = 5) and TRAF3 siRNA (*P* = .0001, *n* = 5) but not IRF7 siRNA (*P* = .13, *n* = 5). Bars represent the median while error bars represent interquartile range. (F,G) Overlay graphs showing expression of TGF‐β1 after treatment with inhibitors of MyD88 and IRAK1. (H) The statistical comparison of intracellular per cell expression of TGF‐β after inhibition of MyD88 and IRAK1 showed similar expression after MyD88 inhibition (*P* = .44, *n* = 5) but lower expression after IRAK1 (*P* = .04, *n* = 5) inhibition. Bars represent the median while error bars represent the interquartile range. (I) ELISA results showed lower similar levels of TGF‐β after MyD88 (*P* = .65, *n* = 5) but lower levels after IRAK1 inhibition (*P* = .035, *n* = 5). Bars represent the median while error bars represent interquartile range. The difference in TGF‐β expression within groups for both siRNA and inhibitor‐treated sets was compared using repeated measures one‐way ANOVA for all comparisons except ELISA where it was compared using Friedman test, while post hoc differences were tested using Dunnett's multiple comparisons test except for ELISA where same was tested using Dunn's multiple comparisons test

Since TGF‐β1 (LAP) is an integral component of FoxP3‐ regulatory T‐like cells, we tested whether an increase in TGF‐β expression in CD4+ T cells post ODN 2216 uptake was dependent on TLR9 signalling. In this context, we inhibited TLR9 signalling at various levels and assessed the protein expression of TGF‐β in ODN 2216 stimulated cells. ODN 2216 stimulation resulted in a clearly visible increase in intracellular TGF‐β expression (Figure 5A), as well as secreted protein levels (Figure 5I). We observed a significant decrease in intracellular expression of TGF‐β in CD4+ T cells, after treatment with TLR9 siRNA (*P* = .0004, *n* = 5) (Figure 5B,E) and TRAF3 siRNA (*P* = .0001, *n* = 5) (Figure 5C,E) but not IRF7 siRNA (*P* = .13, *n* = 5) (Figure 5D,E) as compared to controls. Interestingly, the inhibition of MyD88 did not result in significant decrease in intracellular TGF‐β expression (*P* = .44, *n* = 5) (Figure 5F,H). Inhibition of IRAK1 on the other hand showed a significant decrease in intracellular expression (*P* = .04, *n* = 5) (Figure 5G,H) and secretion (*P* = .035, *n* = 5) (Figure 5I) in CD4+ T cells. ELISA results confirmed that secretion of TGF‐β was not significantly decreased after MyD88 inhibition (*P* > .99, *n* = 5) (Figure 5I) as compared to control cells. These results indicated that the transition of ODN 2216 treated CD4+ T cells towards an anti‐inflammatory phenotype is independent of MyD88.

### 3.7 ODN 2216 uptake‐induced increase in CD4+ T cell proliferation is dependent TLR9 signalling

**FIGURE 6:**
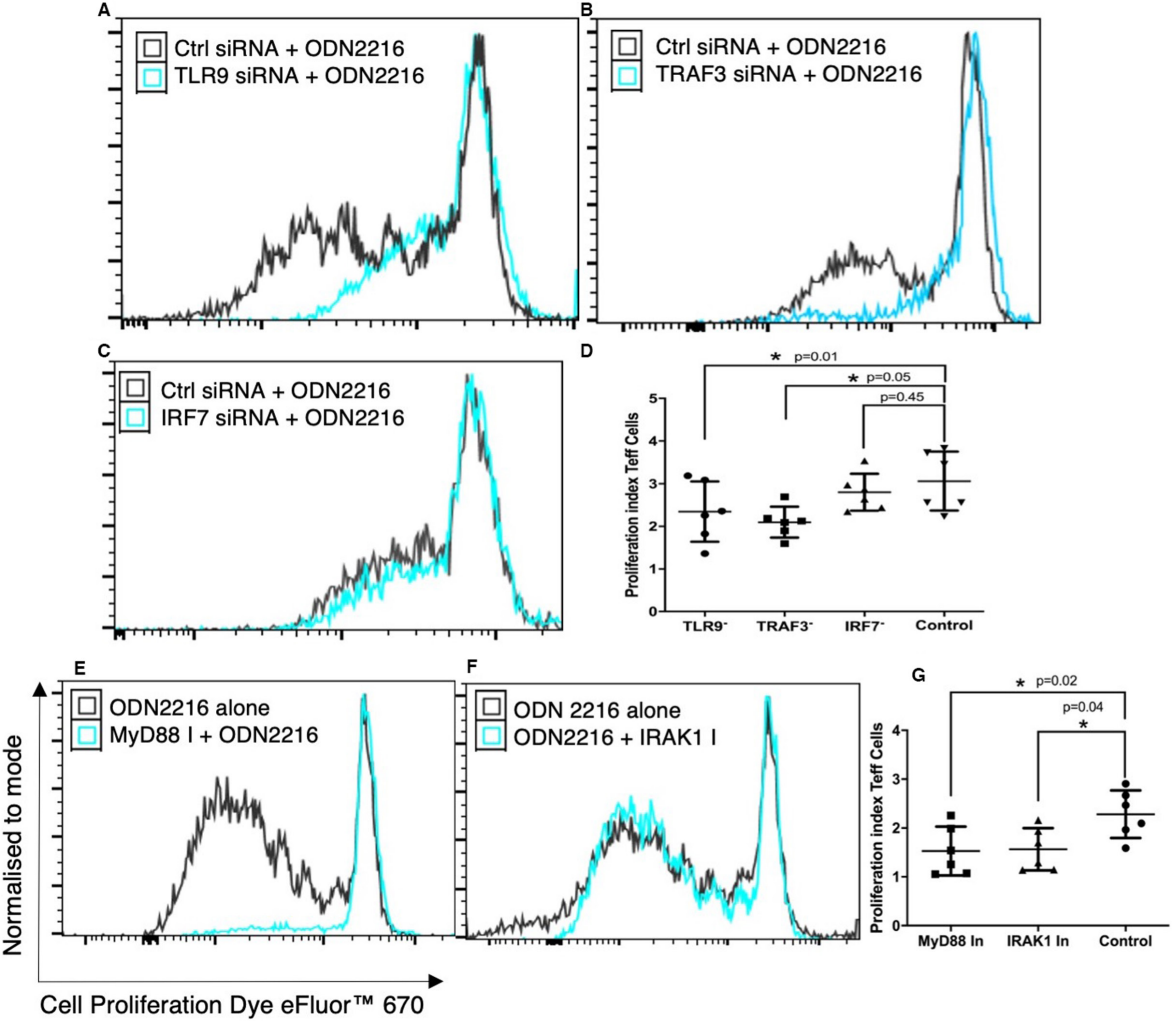
Effect of TLR9 signalling inhibition on the increase in CD4+ T cell proliferation after ODN 2216 ligation. CD4+ T cells were assessed for cell proliferation using efluor670 dye after 3 days of treatment with inhibitors of TLR9 signalling molecules or control conditions. (A‐C) Overlay graphs showing differences in proliferation upon inhibition of TLR9, TRAF3 and IRF7. (D) Statistical comparison of the effect of inhibition on T cell proliferation shows significantly lower proliferation indices in cells silenced with TLR9 siRNA (*P* = .01, *n* = 6), TRAF3 siRNA (*P* = .05, *n* = 6) but not IRF7 siRNA (*P* = .45, *n* = 6). (E,F) overlay of proliferation graphs for MyD88 and IRAK1 inhibition. (G) Statistical comparison showed lower proliferation index after MyD88 (*P* = .02, *n* = 6) and IRAK1 (*P* = .04, *n* = 6) inhibition. The statistical difference was tested using repeated measured one‐way ANOVA. Each dot represents data from one donor in the individual graphs. Post hoc comparisons were made using Sidak's multiple comparisons test

We and others have shown that ODNs increase the proliferation of CD4+ T cells.^22^, ^23^, ^24^ To better understand whether the increase in proliferation upon ODN 2216 exposure is dependent on TLR9 signalling, we measured the effect of ODN 2216 uptake on the proliferation of CD4+ T cells after treating them with inhibitors of TLR9 signalling molecules in independent sets of experiments. There was a significant difference in proliferation indices within groups both siRNA transfected (*P* = .0155, *n* = 6) and inhibitor‐treated sets (*P* = .018, *n* = 6). We observed significantly decreased proliferation indices in cells silenced with TLR9 siRNA (2.30 ± 0.75, *P* = .01, *n* = 6) (Figure 6A,D), TRAF3 siRNA (2.01 ± 0.48, *P* = .05, *n* = 6) (Figure 6B,D) but not IRF7 siRNA (*P* = .45, *n* = 6) (Figure 6C,D) as compared to controls (2.98 ± 0.62) (Figure 6D). In another set of experiments, we observed significantly decreased proliferation in cells treated with MyD88 inhibitor (1.52 ± 0.46, *P* = .02, *n* = 6) (Figure 6E,G) and IRAK1 (1.61 ± 0.69, *P* = .04, *n* = 6) (Figure 6F,G) as compared to control (2.32 ± 0.18) (Figure 6G). Collectively, these findings establish that proliferative responses towards ODNs are dependent on TLR9 signalling and primarily involves TLR9 and MyD88 with feedback dependence on IRAK1 and TRAF3, but not IRF7.

### 3.8 ODN 2216 stimulated CD4+ T cells can suppress the proliferation of untreated CD4+ T cells

**FIGURE 7:**
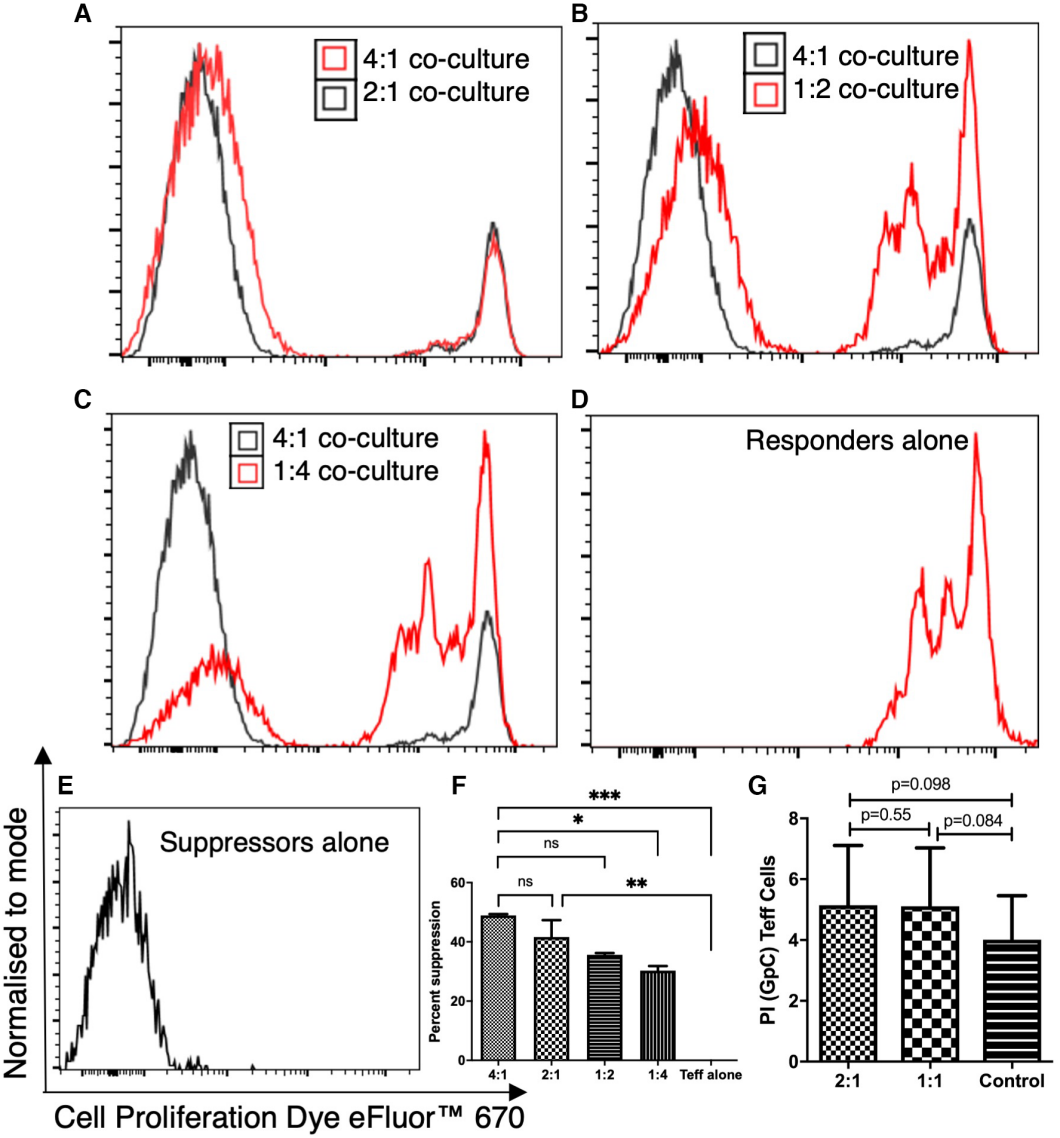
Effect of ODN 2216 stimulated CD4+ T cells on the proliferation of untreated cells. ODN 2216 treated CD4+ T cells (suppressors) were cultured in presence of eFluor 670 labelled untreated CD4+ T cells (responders) to confirm the suppressor phenotype. The comparison of 4:1 ratio with (A) 2:1, (B) 1:2 and (C) 1:4 showed visible differences in proliferation of responders. The peaks on the right‐hand side represent responders, while those on the left side of the histogram depict suppressor cells in figures A‐C. (D) Responder cells alone in T cell optimizer expansion SFM containing IL‐2 and human T cell activator cocktail were used as control. The cells were proliferating in absence of suppressor cells. (E) Suppressors alone occupy the extreme left position on the fluorescence axis. (F) There was a significant difference in percent suppression between different groups (*P* = .0001, *n* = 6). 4:1 ratio showed higher suppression than 1:4 (*P* = .01, *n* = 6). (G) Responder cells on the other hand showed similar proliferation in the presence or absence of suppressor cells stimulated using GpC ODN 2243. The difference in suppression among various groups was tested using the Friedman test, while for post hoc analysis was made using Dunn's multiple comparisons test

In view of the increase in expression of anti‐inflammatory molecules, after ODN 2216 ligation, we assessed the functional impact of TLR9 ligation in CD4+ T cells, using T cell suppression assay. We assessed the ability of ODN 2216 treated suppressor cells to control the proliferation of autologous responder cells. A higher ratio of suppressor cells showed a visible effect on proliferation of responder cells at all ratios namely 4:1, 2:1, 1:2, 1:4 as compared to responders' control (Figure 7A‐E). We observed significant differences in suppression by suppressor cells among different ratios of suppressor:responders (*P* < .0001, *n* = 6). Between group comparison showed significant suppression by 4:1 ratio as compared to 1:4 (*P* = .03, *n* = 6) and Teff alone (*P* = .0006, *n* = 6) (Figure 7F). These observations showed a dose‐dependent suppressive effect by ODN 2216 experienced cells. However, the effect was specific to ODN 2216 stimulated suppressors, as GpC ODN 2243 stimulated T cells failed to show a similar response (Figure 7G). The level of suppression was similar to CD4 + CD25 + Treg induced suppression (*P* = .25, *n* = 3) (Figure S6). Collectively, our observations established the suppressive ability of ODN 2216 treated CD4+ T cells at various ratios.

## 4 DISCUSSION

TLRs are immune receptors predominantly expressed on innate immune cells including monocytes, macrophages and pDCs.^36^ A handful of studies have shown the engagement of TLRs on adaptive immune cells, predominantly B‐lymphocytes.^18^, ^32^, ^37^, ^38^, ^39^ Earlier studies reported the absence of TLR9 in T cells,^39^, ^40^ though it was later discovered that T cells express variable levels of TLR9.^41^, ^42^ The engagement of TLR9 in B cells has been shown to determine class switch recombination.^43^ However, the functions of TLR9 in T cells, primarily CD4 + T cells are not well described. There are a very few reports, which have focused on the dependence of ODN‐induced functions on TLR9 or signalling molecules in T cells.^22^, ^23^, ^24^ Gelman et al^22^ reported that activated CD4+ T cells express TLR9 and that stimulation with CpG DNA increases their survival in a MyD88‐dependent manner. The same group later showed that an increase in proliferation and prevention of anergy depend on the MyD88 and PI3 kinase‐dependent pathway.^23^ However, Landrigan et al^24^ observed that ODN‐induced enhanced proliferation is independent of TLR9 and MyD88. In the wake of these limited and contrasting reports, we planned this study to comprehensively investigate the ligand‐receptor relationship of ODN and TLR9 in CD4+ T cells. We chose to work on ODN 2216, a well‐studied class A CpG ODN and TLR9 agonist shown to induce IL‐10 expression^13^ and decrease IL‐6 expression^14^ in B cells and Treg induction via ligation on pDCs.^15^ We focused on CD4 + CD25‐ T cells rather than the entire CD4+ T cell population to exclude the role of Tregs, since it is known that ODNs promote the survival of Tregs.^25^

Using various approaches, we demonstrated that inhibition of TLR9 and MyD88 resulted in a substantial decrease in the uptake of ODN 2216 in CD4+ T cells, suggesting their role as receptors in the process. We have shown that inhibition of endocytosis using Phenergan or incubation at low temperatures results in abolition of ODN 2216 uptake in CD4 + T cells.^20^ By inhibiting TLR9 using specific siRNA, we observed a more than a 2‐fold decrease in TLR9 protein expression. To the best of our knowledge, we reported for the first time that ODNs are taken up by CD4+ T cells via TLR9 and MyD88. This is in line with previous studies on innate immune cells.^44^, ^45^, ^46^ TLR9 signalling is a well‐defined feedback inhibition controlled pathway in innate immune cells^47^, ^48^ where studies have reported activation of TLR9 signalling upon exposure to ODNs.^49^ Using gene expression array, we could demonstrate that ODN uptake activates TLR9 signalling pathway in CD4+ T cells as well. This activation was delayed and not immediate, very much in line with the available data for B cells.^32^ This delay could be attributed to the evolution of CD4 + T cells; being adaptive immune cells, these cells are expected to have higher activation threshold towards TLR ligands as compared to innate immune cells. Further, the increased expression was specific to ODN 2216 and was not seen in TLR9 antagonist treated or untreated cells. Moreover, binding of CpG is believed to be rate‐limiting step and thus may be subject to regulation by various molecules of TLR9 signalling. Recent studies have also shown a role for various molecules like granulin, DEC‐205 and mannose receptor‐1 in the uptake of CpG ODNs in innate immune cells.^50^, ^51^, ^52^ Since most of these receptors are not yet reported to be expressed in CD4 + T cells, it will be interesting to look at the differences in the uptake of different classes of ODNs in the context of receptors utilized and the differences in the conformation of various ODNs in CD4+ T cells.

Most of the studies on the effect of ODN ligation on sorted T cells have reported an increase in proliferation or the increase in IL‐2 expression.^22^, ^23^, ^24^ However, the dependence of increased proliferation on TLR9 signalling has shown contrasting results on the involvement of MyD88 in this process.^23^, ^24^ We could demonstrate that ODN 2216 uptake resulted in increased proliferative and immunosuppressive capacity and that these changes were dependent on functional TLR9 signalling pathway, not involving IRF‐7. In this context, a previous study has shown that TGF‐β inhibits IRF‐7 induced type‐I interferon synthesis post CpG ODN challenge.^53^ It is also noteworthy that TGF‐β1 has been shown to result in ubiquitination of IRF7 and TRAF6 in Hela cells, which causes lower IFN responses. Previously, non‐CpG DNA has been shown to induce Th2 type of immune responses^54^ and the differences in responses between CpG and non‐CpG DNA may be attributed to differences in methylation, conformation or size of the molecules. This study showed that nucleic acid sensing in T cells was independent of known DNA sensors like absent in melanoma 2 (AIM2) or stimulator of IFN genes (STING).^54^ It will be interesting to look at the cellular receptors involved in uptake of ODNs and to test differences in receptors for CpG and non‐CpG DNA and their physiological relevance.

Studies have shown induction of Tregs in response to ODN‐induced activation of pDCs.^15^, ^55^ We found that ODN 2216 ligation resulted in an increase in TGF‐β1, CTLA4 and IL‐10 but not FoxP3 expression in these cells, which possibly contributed to the suppressive potential of these cells. The level of suppression observed with ODN 2216 treated CD4+ T cells was similar to those with CD4 + CD25 + T cells (Tregs) in this and, also to controls in our previous study.^31^ However, the stability of this suppression and other functional aspects in different disease settings needs to be explored further. These experiments confirmed that ODN 2216 exposure of CD4+ T cells result into an anti‐inflammatory phenotype, which was similar to Th3 type of T cells. The molecular mechanism of such responses including epigenetic control by exogenous CpG ODNs is still far from clear and needs further studies. To the best of our knowledge, this is the first study demonstrating immunomodulation of CD4+ T cells following ODN uptake, which is independent of APCs.

Our study can have clinical relevance in inflammatory diseases involving CD4+ T‐cell functions as a major part of the immunopathology of the disease. In this context, we recently observed that ocular infiltrating CD4+ T cells in patients with tubercular uveitis show lower uptake of TLR9 ligand than CD4+ T cells in peripheral blood, indicative of the role of local microenvironment in the modulation of T cell response to TLR challenge.^26^ In another study, we observed that TLR9 ligand challenge in sorted CD4+ T cells results in increased proinflammatory cytokine response, correlating with ocular inflammatory activity in these patients.^21^ Few earlier studies, in infectious and autoimmune diseases have also indicated altered T‐cell responses to TLR ligand challenges.^56^, ^57^ Our results thus provide a mechanism of action of these ligands in such conditions of clinical relevance. Similar expression of TLR9 in CD4+ T cells and B cells indicate that even CD4+ T cells could play a role by involving TLR9 under autoinflammatory and autoimmune disease conditions. It would be specially interesting to look at the role of TLR9 in CD4+ T cells in diseases like anti‐neutrophil cytoplasmic antibody associated vasculitis, where TLR9‐related responses have been shown to be dysregulated in circulating dendritic cells.^58^ TLR9 ligands have been shown to be important constituents of neutrophil cytoplasmic traps (NETs) and activation of TLR9 has been implicated in the induction of NETs.^59^ These NETs act as a source of autoantigens in the disease and are also associated with granuloma formation. The responsiveness of CD4+ T cells to TLR9 ligands in the microenvironment of granuloma may play a significant role in the inflammatory response. Recently, a study has shown induction of IL‐10 producing Tregs and wound healing macrophages in mouse models of ulcerative colitis in a TLR9‐dependent manner.^60^ Our study shows that similar effects can also be observed in pure cultures of CD4 + T cells and thereby CD4 + T cells can be modulated by innate immune triggers. This becomes especially important in diseases involving inflammation of local sites for example ocular tuberculosis, where some patients demonstrate a biased infiltration of T cells into the local site.^26^ There is accumulative evidence suggesting the interactions between DNA and CD4 + T cells. Recent studies have shown the conversion of conventional CD4 + T cells into Tregs due to exposure of self‐DNA in Listeria infection^61^ and self‐DNA induced stimulation of activated CD4 + T cells.^62^ These studies point towards some of the implications of this unexplored interaction of adaptive immune cells and innate immune triggers in health and disease.

Despite establishing the role of TLR9 signalling in CpG ODN 2216‐induced responses in CD4+ T cells, our study had a few limitations. We could not investigate whether other classes of ODNs and non‐CpG DNA can induce similar changes in CD4+ T cells, nor did we investigate the expression of other transcription factors such as T‐bet or RORγT in ODN 2216 treated CD4+ T cells. Nonetheless, our study could establish the functional context of TLR9 signalling in CD4+ T cells. It will be interesting to look at role of TLR9‐mediated functions in the context of disease pathology in diseases like systemic lupus erythematosus, where anti‐DNA responses constitute a central part of disease pathology. To conclude, our study emphasizes that in addition to classical innate immune cells, CD4 + T cells can also be a direct target of TLR ligands. This can have far‐reaching implications for modulation of T cell responses in various infectious and inflammatory diseases. Our findings thus, pave the way for future research to explore direct modulation of adaptive immune cells, using innate immune triggers.

## AUTHOR CONTRIBUTIONS

NS and AG provided overall guidance in the study. RKS and NS conceptualized the study. RKS, NS, SS and PJ planned the experiments. RKS performed the majority of bench work with assistance from JS, RK and AP. Results were analysed and interpreted together by RKS, NS, SS and PJ. Manuscript was written by RKS and NS followed by evaluation and editing by all the authors.

## FUNDING INFORMATION

Centre of Excellence, Advance Eye Centre, PGIMER funded by Department of Biotechnology (DBT), Government of India, New Delhi for support (Grant No. BT/01/CEIB/11/02) (awarded to AG and NS) and Indian Council of Medical Research for providing fellowship to RS (3/1/3/JRF‐2012/HRD‐87). This study was supported in part by the National Institutes of Health/Fogarty International Centre training grant D43 TW009588 (awarded to SL), and the 1R01NS097147 grant (awarded to PJ).

## CONFLICT OF INTEREST

The authors declare no conflict of interest.

## ETHICAL APPROVAL

The study was duly approved by PGIMER Chandigarh institute ethics committee (No's PGI/IEC/2011/532‐533/24/11/11 and NK/1385/PhD/2484/07/09/17). This study was executed in accordance with the recommendations of the Institutional ethics committee of PGIMER.

## Supporting information


Appendix S1

## Data Availability

The data that support the findings of this study are available from the corresponding author upon reasonable request.
