# Supplementary material for: TLR9 signalling activation via direct ligation and its functional consequences in CD4 + T cells
Source: Scand J Immunol. Author manuscript; Available in PMC 2023 Nov 1. (PMC9788197; doi:10.1111/sji.13214)
Supplement: Supplementary dataset [file NIHMS1833005-supplement-Supplementary_dataset.docx]

**TLR9 signaling activation via direct ligation and its functional consequences in CD4+T cells**

Ravi Kumar Sharma^1,2,#,*^, Jyoti Sharma^1^, Rajendra Kumar^3,€^, Darshan Badal^4^, Ajinkya Pattekar^2^, Shobha Sehgal^5^, Amod Gupta^1^, Pooja Jain^2^ and Naresh Sachdeva^4*^

^1^Advanced Eye Centre, Post Graduate Institute of Medical Education and Research (PGIMER), Chandigarh, India.

^2^Department of Microbiology and Immunology and the Institute for Molecular Medicine and Infectious Disease, Drexel University College of Medicine, Philadelphia, PA, USA.

^3^Division of Biological Sciences, Indian Institute of Science Education and Research, Mohali, Punjab, India.

^4^ Department of Endocrinology, Post Graduate Institute of Medical Education and Research (PGIMER), Chandigarh, India.

^5^ Department of Immunopathology, Post Graduate Institute of Medical Education and Research (PGIMER), Chandigarh, India.

***Correspondence:** [naresh_pgi@hotmail.com](mailto:naresh_pgi@hotmail.com), [Tel:](Tel:-) +91-172-2755282

***Co-Correspondence**: ravi.kumar@ki.se, Tel: +46769518010

^#^Present address: Division of Rheumatology, Department of Medicine, Karolinska Institutet, Solna, Sweden

^€^ Present address: Oncology, Johns Hopkins University School of Medicine, Maryland, USA

**!**A portion of this work has been presented as Oral talks during IMMUNOLOGY 2017^TM^, the American Association of Immunologists (AAI) Annual Meeting, May 12-16, Washington D.C., USA and at the 5^th^ European Congress of Immunology 2018, September 2-5, Amsterdam, The Netherlands.

**SUPPLEMENTARY DATA**

**Supplementary methods:**

**Gating strategy for flow cytometry:** Magnetically sorted CD4+CD25-T cells were assessed for purity (in representative samples) by gating on the basis of expression of CD3, CD4 and lack of CD25 and FoxP3 on the day of sorting **(Supp.Fig.1)**. The same gating strategy (Supp.fig.1a-b) was used to gate CD4+T cells in (Fig 2,3,5,6,7), while for figure 4, CD4+T cells were further divided into CD4+CD25+ and CD4+CD25- T cell populations.

**Table 1: List of genes analysed in real time RT-PCR array**

|  |  | 1 |  | 2 |  | 3 | 4 |
| --- | --- | --- | --- | --- | --- | --- | --- |
| A |  | TLR9 |  | CHUK (IKK-α) |  | IRF3 | ACTB |
| B |  | MYD88 |  | IKBKB |  | IRF7 | RTC |
| C |  | IRAK4 |  | IKBKE |  | TBK1 | PPC |
| D |  | IRAK1 |  | TAB1 |  | NFKB1 | GDC |
| E |  | TRAF6 |  | TAB2 |  | RELA |  |
| F |  | UBE2N |  | MAP2K4 |  | CREB1 |  |
| G |  | TRAF3 |  | MAP2K7 |  | TANK |  |
| H |  | NR2C2 |  | MAPK8IP3 |  | GAPDH |  |

**Table 2: Sequences of primers used for confirmation of RT PCR-array results**

| S.No | Gene | Forward Primer 5’-3’ | Reverse Primer 5’-3’ |
| --- | --- | --- | --- |
| 1. | TLR9 | CCACCCTGGAAGAGCTAAACC | GCCGTCCATGAATAGGAAGC |
| 2. | MyD88 | GAGCGTTTCGATGCCTTCAT | GTTTGTCTGTTCCAGTTGCCG |
| 3. | IRAK1 | CGAGGAGTACATCAAGACGGG | TGTGCTCTGGGTGCTTCTCA |
| 4. | IRAK4 | TAGTTCGGCTGGTTCTTCTGTC | CATTGAGGCAGCGCACATA |
| 5. | TRAF3 | GCTGTCCTGACAGAAGAGAAC | TCAGGGACAAAAACTGGCGT |
| 6. | TRAF6 | TCTGTGTCCGTCCTCTACCA | GAGCACACAAAGAAAGCTGGG |
| 7. | IRF7 | CGCCACTGTTTAGGTTTCGC | GCTGCCTCGGTATGGATCTC |
| 8. | GAPDH | GGTGTGAACCATGAGAAGTATGA | GAGTCCTTCCACGATACCAAAG |

**Supplementary Figures:**

**
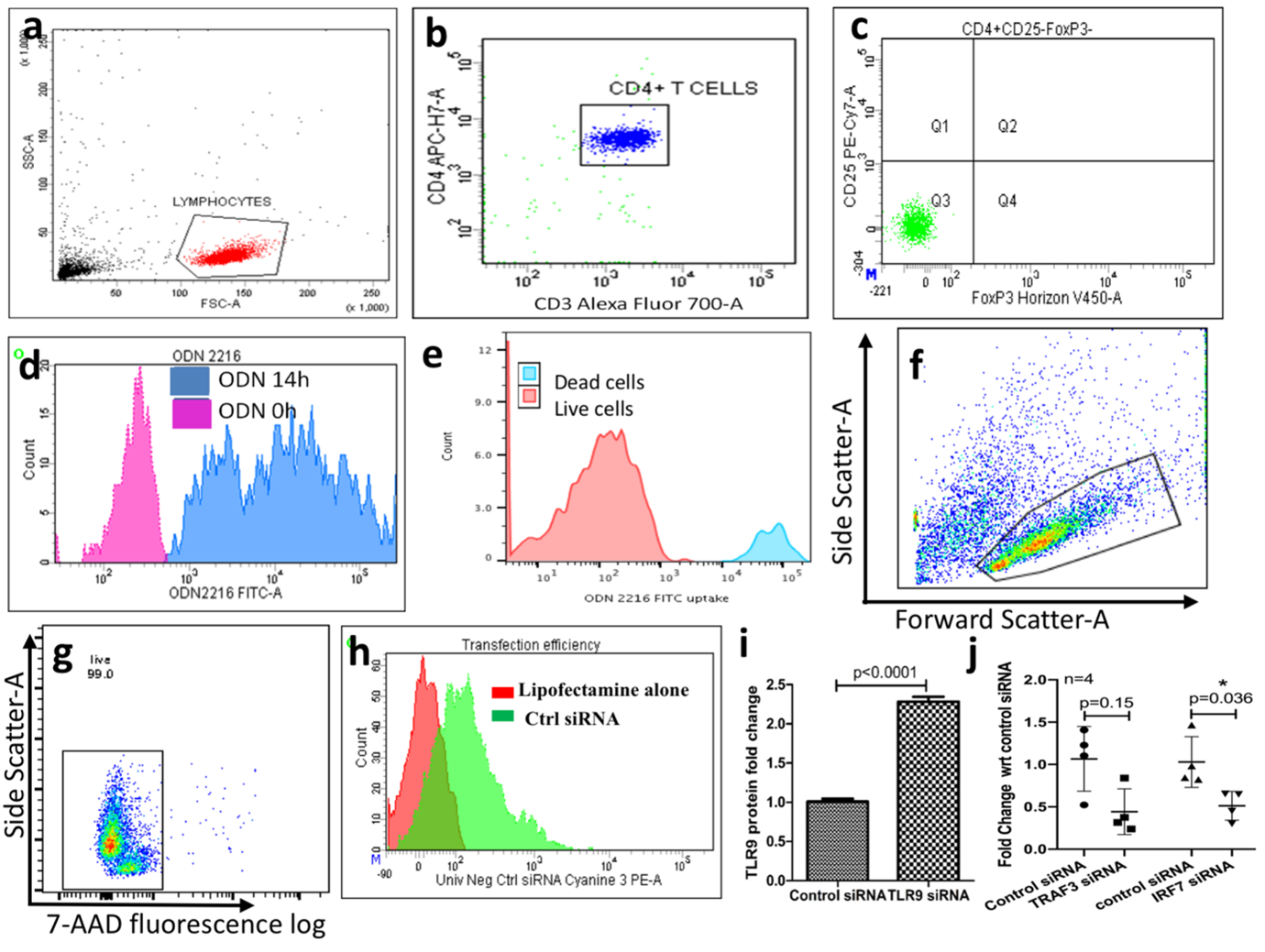
**

**Supp Fig. 1: Teff cell sorting and localization of ODN 2216 in CD4+ Teff cells.** CD4+Teff cells were isolated from PBMCs and characterized using flow cytometry. (a) Isolated cells were gated on forward and side scatter (b) CD3+CD4+ T cells were gated from the parent gate. Purity of CD3+CD4+ T cells was > 98%. (c) CD3+CD4+T cells were then evaluated for expression of CD25 and FoxP3 in representative samples to confirm the effector phenotype i.e. CD3+CD4+CD25-FoxP3-. (d) Teff cells were treated with ODN 2216 FITC (500 ng/ml) and incubated at 37 °C for 14 hrs to assess the shift in fluorescence in FITC channel which was used as a measure of ODN 2216 uptake in Teff cells. (e) To distinguish the uptake pattern of ODN 2216 in dead cells, we also assessed the uptake levels and dead cells showed extremely high levels of ODN 2216, probably due to stickiness. (f) A representative flow cytogram showing selection of live cells using tight forward and side scatter gating. (g) The tightly gated cells were further gated for live cells on the basis of 7-AAD fluorescence in PerCP channel. On an average, 98-99% of gated cells were negative for 7-AAD fluorescence (live). (h) Transfection using control siRNA resulted in clear shift in fluorescence in PE channel as compared to those transfected using transfection reagent alone. (i) Silencing using TLR9 siRNA resulted in more than two-fold decrease in TLR9 MFI as compared to those treated using control siRNA. (j) Transfection using TRAF3 and IRF7 siRNA also resulted in more than two-fold decrease in mRNA expression of respective genes in sorted CD4+ Teff cells. Cells transfected using universal negative control siRNA were used as control, while GAPDH was used as endogenous control in these experiments.


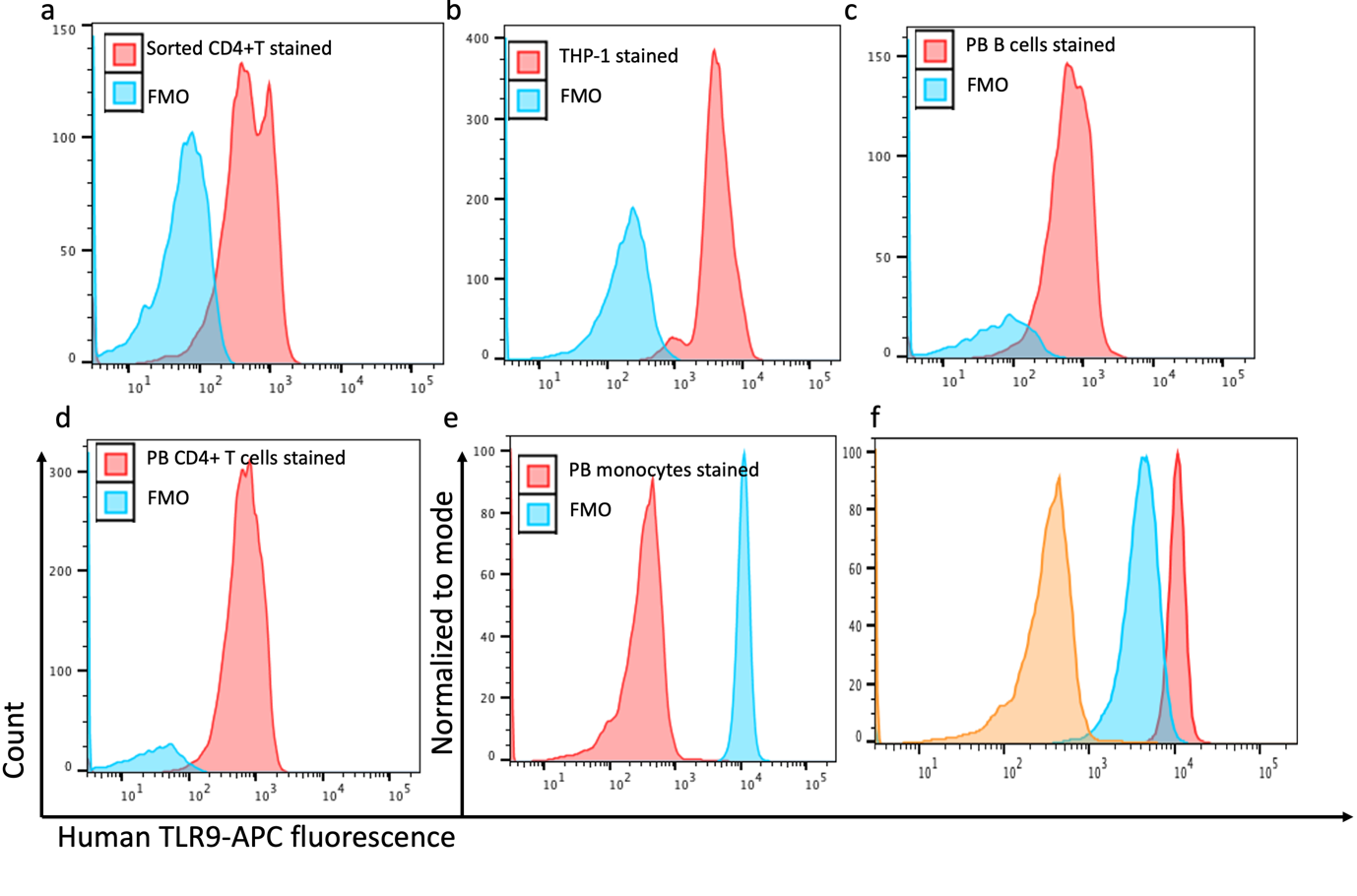


**Supp. Fig. 2: Comparative expression of TLR9 in various cell types.**

We performed a comparative study to assess the relative expression of TLR9 in various cell types. (a) Flow cytometry sorted CD4+ T cells showed clear resolution of TLR9 expressing cells from the background. (b) THP-1 cell line (human monocyte cell line) showed clearly positive staining for TLR9. (c-e) Similarly, we also assessed TLR9 expression pattern in B cells, CD4+ T cells and monocytes directly in peripheral blood (f) Overlay of relative expression of TLR9 between CD4+T cells (aqua) and monocytes (red), where fluorescence minus one is shown in wheat colour.


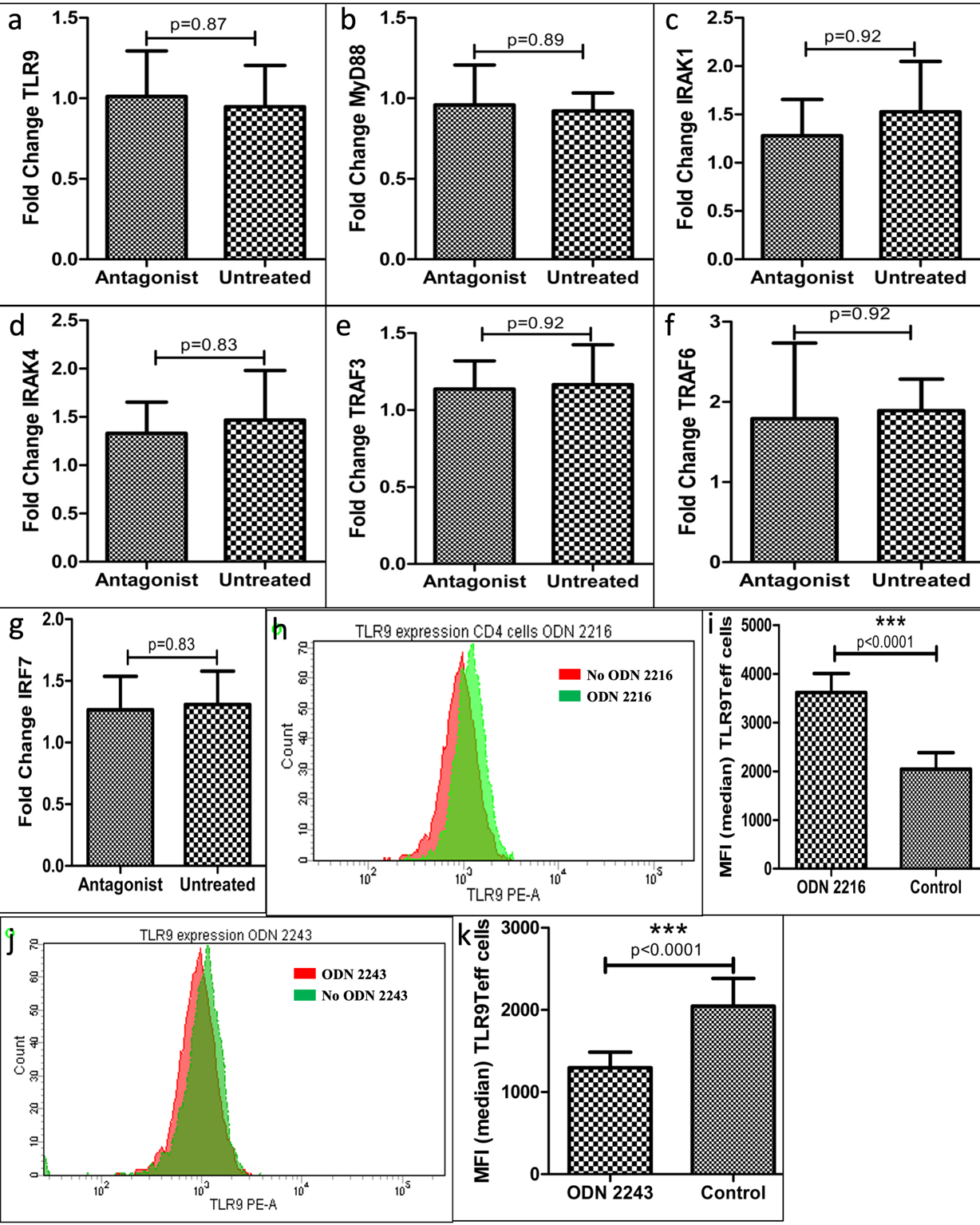


**Supp. Fig. 3: The effect of ODN 2216 uptake on activation of TLR9 signaling.**

To test whether the effects of ODN 2216 ligation on increase in expression of genes of TLR9 signalling are specific, we treated Teff cells with TLR9 antagonist ODN TTAGGG and evaluated the expression of genes involved. We observed similar relative expression of (a) TLR9 (p=0.87), (b) MyD88 (p=0.89), (c) IRAK1 (p=0.92), (d) IRAK4 (p=0.83), (e) TRAF3 (p=0.92), (f) TRAF6 (p=0.92) and (g) IRF7 (p=0.83) in antagonist treated and untreated Teff cells. (h) ODN 2216 stimulation in CD4+ Teff cells resulted in a visible shift in fluorescence intensity of TLR9. (i) The increase in TLR9 MFI was significantly higher in CD4+Teff cells treated with ODN 2216 as compared to untreated control cells (p<0.0001). (j) GpC ODN 2243 (control ODN) stimulation, on the other hand did not increase the expression of TLR9. (k) TLR9 MFI was significantly lower after ODN 2243 stimulation as compared to control cells (p<0.0001).


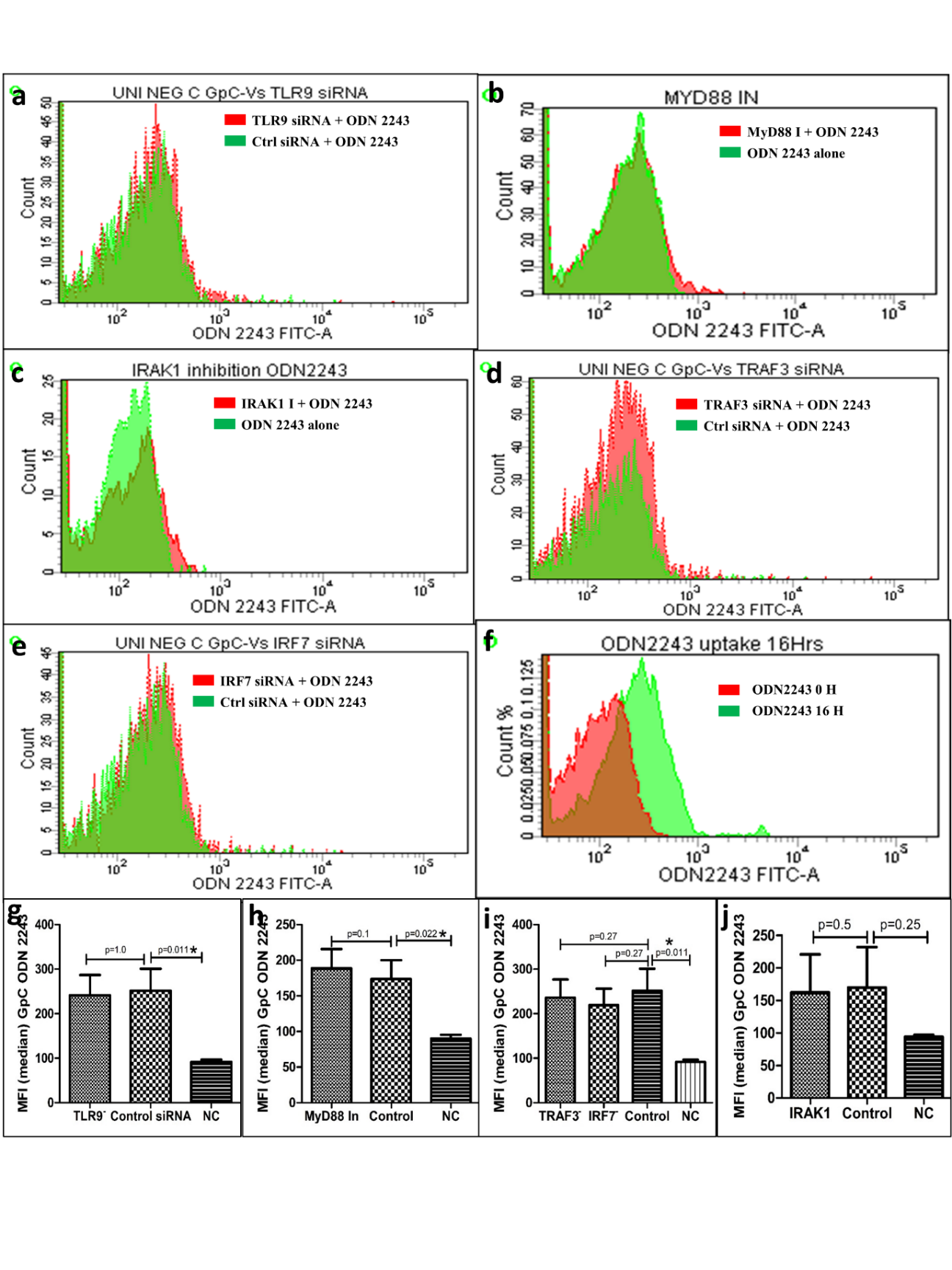


**Supp. Fig. 4: The effect of TLR9 signalling inhibition on ODN 2243 in CD4+ Teff cells.** GpC ODN 2243 was used as a control to assess whether the effects are specific to CpG ODN 2216. To test whether uptake of ODN 2243 could be influenced by TLR9 signalling, we inhibited various molecules of TLR9 signalling pathway and assessed the uptake of ODN 2243. The fluorescence in FITC channel was compared between inhibitor treated cells and control cells. There was no visible shift in fluorescence in FITC channel after inhibition of (a) TLR9, (b) MyD88, (c) IRAK1, (d) TRAF3, (e) IRF7. (g) ODN 2243 uptake was clearly visible in CD4+ Teff cells as compared to untreated cells. Statistically, (h) the uptake of ODN 2243 was similar between cells transfected with TLR9 siRNA and control siRNA (p=1.0). (i) The inhibition of MyD88 did not affect the uptake of ODN 2243 (p=0.1). (j) Transfection of CD4+Teff cells with TRAF3 or IRF7 siRNA, showed similar uptake of ODN 2243 as in those transfected using control siRNA (p=0.27). (k) The inhibition of IRAK1 also showed similar uptake of ODN 2243 as that in control cells (p=0.5).


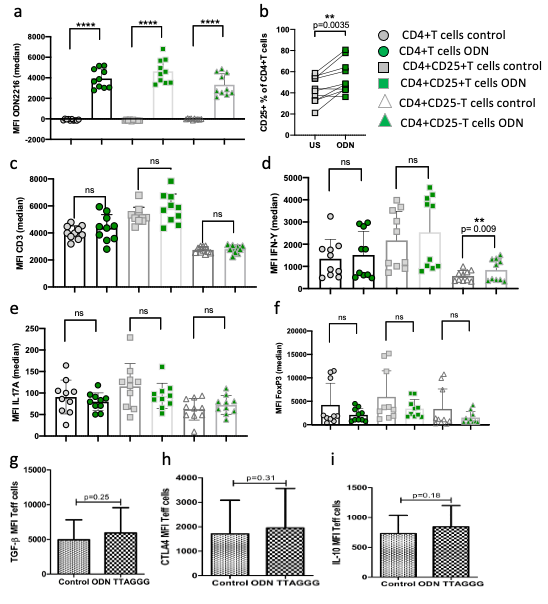


**Supp. Fig. 5: The effect of ODN2216 stimulation on phenotype of CD4+ Teff cells.** (a) ODN 2216 stimulation resulted in significant uptake of ODN 2216 in total CD4+T cells, as well as CD4+CD25+ and CD4+CD25- T cell subsets (paired t test). (b) Proportion of CD4+CD25+T cells after ODN 2216 stimulation was compared using paired t test. (c-f) MFI of CD3, IFN-γ, IL-17A and FoxP3 was compared between groups using paired t-test. (g-i) MFI of TGF-β, CTLA4 and IL-10 after stimulation with ODN TTAGGG was compared with control using paired t-test.


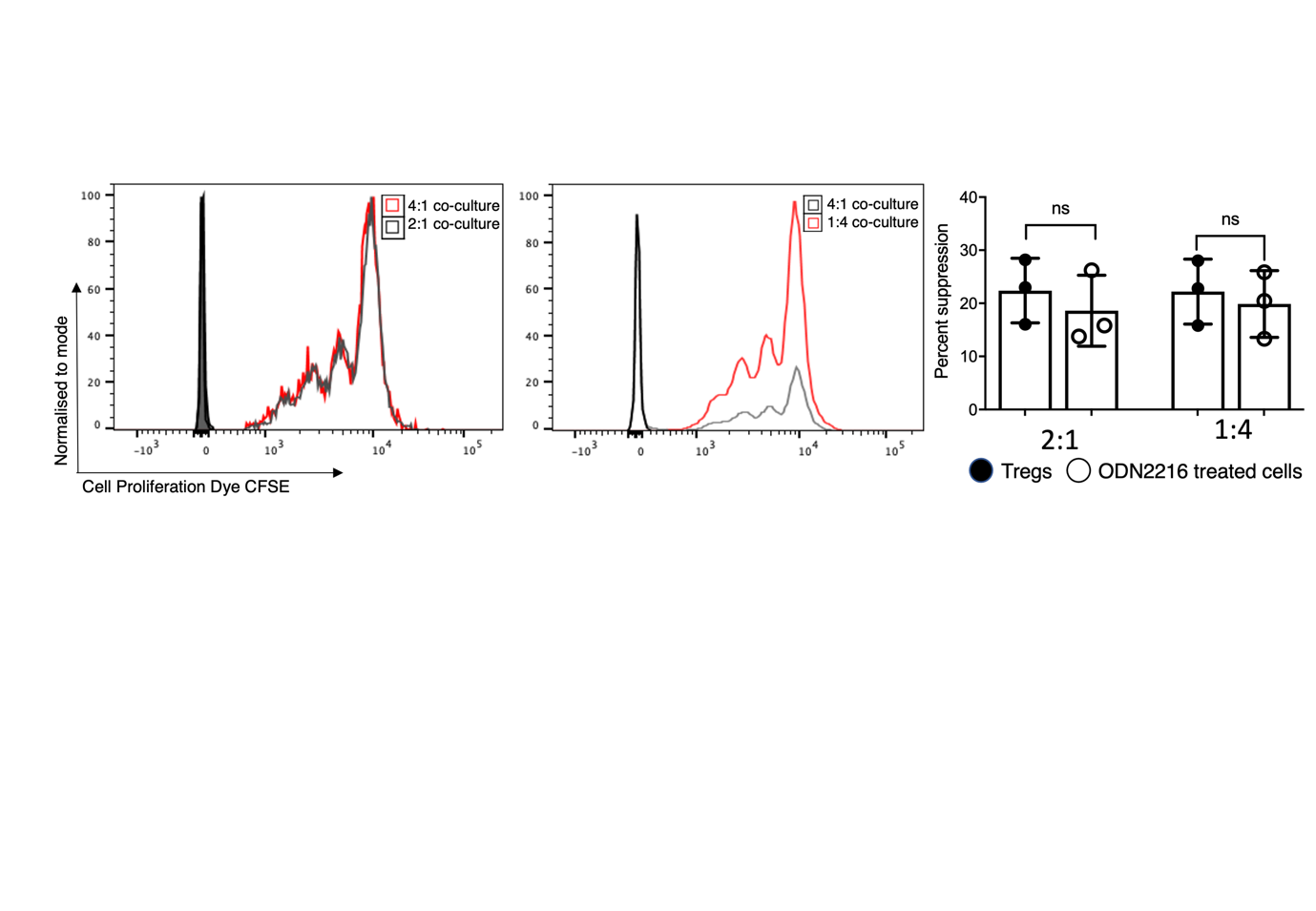


**Supp.Fig.6:** In a few subjects (n=3), we tested suppression by conventional Tregs, where magnetically sorted CD4+CD25+T cells (suppressors) were co-cultured with CD4+CD25-T (responder) cells. The first two plots show proliferation of responder cells at 4:1-2:1 and 4:1-1:4 ratios (Tregs: T responder cells). Statistically, the percent suppression by Tregs and ODN2216 treated cells at same ratios didn’t show any difference in their suppressive ability (p= 0.25).
